# Protective effects of polysaccharide SH-P-1-1 isolated from *Phellinus igniarius* on hyperuricemia and gout via intestinal microecosystem regulation

**DOI:** 10.3389/fnut.2025.1717561

**Published:** 2025-12-19

**Authors:** Yanan Wang, Xinyi Qian, Lingzhi Chen, Ling Yang, Zhenjiang Zhang, Zhilong Qu, Yuhan Yang, Yihao Li, Zaizhong Ni, Ying Shao, Anhui Chen

**Affiliations:** 1 College of Food and Bioengineering, Xuzhou University of Technology, Xuzhou, Jiangsu, China; 2 Jiangsu Key Construction Laboratory of Food Resource Development and Quality Safe, Xuzhou University of Technology, Xuzhou, Jiangsu, China; 3 School of Biotechnology, Jiangnan University, Wuxi, Jiangsu, China; 4 Anhui Yanhuang Biotechnology Co., Ltd., Hefei, Anhui, China

**Keywords:** *Phellinus igniarius*, polysaccharide, structural characterization, hyperuricemia, gout, intestinal microecosystem

## Abstract

**Introduction:**

Although *Phellinus igniarius* has been shown to treat hyperuricemia (HUA) and gout, a self-inflammatory disease caused by purine metabolism disorders, the underlying mechanisms remain unclear. Polysaccharides are among the main components of *P. igniarius* with anti-inflammatory and immunoregulatory properties.

**Methods:**

Here, we examined the therapeutic effects of polysaccharide SH-P-1-1 isolated from *P. igniarius* on HUA and gout, and explored the underlying mechanism, focusing on gut microbiota and metabolite regulation.

**Results:**

SH-P-1-1 significantly decreased uric acid and creatinine levels, reduced xanthine oxidase and adenosine deaminase activities, alleviated kidney damage, and reduced urate deposition in joints in model rats. It increased the abundance of *Blautia* and *Muribaculaceae* but reduced that of the *Lachnospiraceae_NK4A136_group*, *Lactobacillus*, and *Turicibacter*. SH-P-1-1 also significantly modulated the metabolic profile and was closely related to some metabolic pathways, such as tryptophan metabolism, relevant to HUA and gout. The beneficial activities of SH-P-1-1 correlated with gut microbiota composition and differential metabolites, including short-chain fatty acids.

**Discussion:**

Overall, this study demonstrates the potential of SH-P-1-1 as a natural supplement for preventing and managing HUA and gout.

## 1 Introduction

Gout is an auto-inflammatory disorder induced by chronic HUA due to disturbance in purine metabolism (1). It manifests as a gradual deposition of uric acid (UA) salt crystals in joints and surrounding tissues, triggering an innate immune response while inducing stressful arthritis (2, 3). Patients with gout typically present with severe pain, joint swelling and redness, and joint damage, together with serious complications, such as chronic kidney disease, and several metabolic diseases (4). Currently, many drugs used in the clinic, such as allopurinol, benzbromarone, febuxostat, and colchicine, can effectively lower the UA content. However, long-term utilization of such drugs is usually associated with different toxic adverse reactions (5–7). Therefore, the exploration and development of low-toxicity, safe, and effective drugs with natural active ingredients for the management of HUA and gout have received wide attention.

*Phellinus igniarius* is a valuable edible medicinal fungus used to treat various diseases (8, 9). It can effectively alleviate HUA and gout; however, its specific active ingredients are unknown (10–12). Polysaccharides are among the main components of *P. igniarius*, which show distinct bioactivities, including antitumor, hypoglycemic, antioxidative, and hepatoprotective effects (13, 14). Moreover, this fungus has received extensive attention in the fields of regulation of inflammation and immune function. Polysaccharides isolated from *P. igniarius* YASH1 stimulated interleukin-2 (IL-2), interleukin-6 (IL-6), and interferon-*γ* (IFN-γ) production within immune cells and enhanced immunity in immunocompromised mice (8). *P. igniarius* polysaccharides could improve the imbalance in inflammatory factors and alleviated symptoms in mice with dextran sodium sulfate-induced colitis (15). They displayed significant immune-adjuvant activity by elevating the phosphorylation of proteins in the nuclear factor kappa-B (NF-κB) and mitogen-activated protein kinase (MAPK) pathways and increasing the MyD88- and TRIF-dependent secretion of cytokines (16). Moreover, polysaccharide extracted from *P. igniarius* significantly inhibited the activity of xanthine oxidase (XOD), and its expression mediated by peroxisome proliferator-activated receptor *α* (PPARα) in the liver, indicating a decrease in uric acid production (17). The XOD is widely distributed in mammalian tissues and secretions, and has a variety of biological functions including in innate immunity and anti-inflammatory activity (18, 19). Therefore, *P. igniarius* polysaccharides can improve and treat HUA and gout by reducing inflammation and regulating immune function.

The gut microbiota plays an essential role in modulating UA metabolism via the gut–kidney axis, which is tightly associated with the onset and development of gout (20). Alterations in the abundance and composition of the gut microbiota can affect UA and purine metabolism pathways, underscoring the potential of the intestinal flora as a target for preventing and treating gout (1). Patients with gout exhibit a marked increase in the abundance of *Bacteroides caccae* and *B. xylanisolvens* and a decrease in *Faecalibacterium prausnitzii* and *Bifidobacterium pseudocatenulatum* (21). *Lactobacillus* and *Pseudomonas* degrade UA into urea by secreting uricase, allantoinase, and allantoicase (22). Metabolic dysfunction of the intestinal microbiota can affect serum UA levels by influencing host metabolite levels (23). Bacterial genera, such as *Lactobacillus*, *Roseburia*, and *Akkermansia*, can also promote the degradation and excretion of UA in the intestinal tract by producing short-chain fatty acids (SCFAs) (24). SCFAs, such as acetate, butyrate, or propionate, produced by the intestinal microbiota, were positively correlated with the effectiveness of HUA treatment in mice (20). Therefore, targeted regulation of the abundance and composition of the gut microbiota and the associated metabolites can help treat HUA and gout.

Polysaccharides that modulate microbial composition and metabolite generation can be used to manage gout. *Sphacelotheca reiliana* polysaccharides alleviated gut microbiota dysfunction and decreased serum metabolite levels in the inflammatory pathways related to kidney injury and gout (25). A novel polysaccharide from *Enteromorpha prolifera* significantly increased the proportion of *Alistipes* and *Parasutterella*, which are negatively correlated with increased UA levels (26). Therefore, whether *P. igniarius* polysaccharides alleviate gout by modulating gut microbiota and their mechanisms needs further investigation.

Accordingly, this current study speculated that SH-P-1-1 can reshape the gut microbiota, enhance the production of beneficial metabolites, including SCFAs, thereby inhibiting XOD activity, reducing the level of UA, and alleviating HUA and gout, finally by reducing inflammation and regulating immune function. To verify this hypothesis, a rat model with HUA and acute gouty arthritis phenotypes was used to analyze the protection rendered by *P. igniarius* polysaccharides against these conditions. Moreover, the relationship between the intestinal microbiota and metabolites, and the therapeutic effects of *P. igniarius* polysaccharides were evaluated, and the mechanism underlying the improvement of HUA and gout by these polysaccharides was explored.

## 2 Materials and methods

### 2.1 *Phellinus igniarius* polysaccharide extract preparation

*Phellinus igniarius* polysaccharides were separated and purified as described previously (27). Briefly, *P. igniarius* powder, obtained after crushing and sieving, was suspended in distilled water (powder:water = 1:20) and heated for 3 h in a water bath set at 85 °C. Thereafter, this mixture was filtered, and the filtrate was concentrated via rotary evaporation (SENCO, China). The concentrated filtrate underwent ethanol precipitation and deproteinization using the Sevag method. DEAE-52 (2.6 cm × 30 cm; Henghuibio, China) and Sephadex G-100 (1.5 cm × 80 cm; Yuanye, China) columns were used for purifying the crude polysaccharides SH-Ps.

### 2.2 Molecular weight determination

The polysaccharide extract (5 mg) was mixed with NaNO_3_ (0.1 mol/L, Sinopharm, China) containing 0.02% NaN_3_ (Sinopharm, China). After filtering through a 0.45 μm pore-size filter, the polysaccharide weight was measured using a DAWN HELEOS-II laser photometer (Wyatt Technology Co., USA) equipped with two tandem columns (300 mm × 8 mm, Shodex OH-pak SB-805 and 803; Showa Denko K.K., Japan).

### 2.3 Monosaccharide composition

Approximately 5 mg of polysaccharide was hydrolyzed with 1 mL of trifluoroacetic acid (2 mol/L, ANPEL, China) at 121 °C for 2 h. The hydrolysate was dried under nitrogen, washed with methanol (ANPEL, China), and subjected to high-performance anion-exchange chromatography on a CarboPac PA-20 anion-exchange column (3 mm × 150 mm; Dionex, USA) with pulsed amperometry detection (PAD, Dionex ICS 5000 + system) to determine its monosaccharide composition.

### 2.4 Fourier transform infrared (FT-IR) spectroscopy

Polysaccharides (2 mg) were mixed with KBr powder (200 mg) and pressed into 1 mm thick pellets. The FT-IR spectra of polysaccharides were obtained in the 4,000–400 cm^−1^ range using an FT-IR spectrometer (Nicolet iZ-10, Thermo Nicolet, USA).

### 2.5 Scanning electron microscopy (SEM)

Freeze-dried polysaccharide samples (5 mg) were sprayed with gold powder using an ion sputtering instrument for 40 s. The gold-coated samples were placed in the observation chamber of a scanning electron microscope (Nova nanoSEM 450, FEI, USA) for observing their surface microstructure at an acceleration voltage of 5 kV.

### 2.6 Ultraviolet–visible (UV–vis) spectroscopy

Polysaccharides were added to distilled water at 5 mg/mL. The UV–vis spectra of the polysaccharide solution were acquired at 200–400 nm using a UV spectrophotometer (UV1900PC, HOTTIE LAB, China).

### 2.7 Methylation analysis

Polysaccharides (3 mg) were added to 500 μL of dimethyl sulfoxide (Sigma, USA), and 1 mg NaOH (Sinopharm, China) was added to the solution. The polysaccharides in the solution were methylated with 50 μL methyl iodide (CNW, China) for 1 h. The methylated products were added to 100 μL trifluoroacetic acid (2 mol/L, CNW, China) and the mixture was incubated for 1.5 h at 121 °C. The samples were then reduced with 50 μL of NaBD_4_ (1 mol/L, Sigma, USA) followed by acetylation by mixing with 250 μL of acetic anhydride (Sigma, USA) and incubating at 100 °C for 2.5 h. The products were dissolved in CH_2_Cl_2_ (CNW, China) and analyzed using an Agilent 6890A-5977B gas chromatograph-mass spectrometer (Agilent Technologies Inc. USA) equipped with an Agilent TG-200 chromatographic column (30 m × 0.25 mm × 0.25 μm, SGE, Australia).

### 2.8 Animals treatment

Specific pathogen-free male Sprague–Dawley (SD) rats (8 weeks old, 220 ± 20 g) were acclimatized for 1 week in a climate-controlled facility (12 h light/dark cycle, 50 ± 10% relative humidity, 22 ± 2 °C) with unrestricted access to food and water. All the rats were fed a regular diet. They were later randomized into a normal (Con, *n* = 5) or a model (*n* = 25) group. The model-group rats were intragastrically administered potassium oxonate (300 mg/kg, Yuanye, China) and adenine (50 mg/kg, Rhawn, China) once daily for 14 consecutive days, whereas the normal-group rats were administered an identical volume of vehicle. On day 14, the model-group rats were injected with 50 μL of a 25 mg/mL suspension of monosodium urate crystals (Rhawn, China) into the right ankle, whereas the normal-group rats were administered an identical volume of saline. The model rats were randomized into five groups: model control (Mod; administered saline, *n* = 5) group; positive control (All; gavage 10 mg/kg allopurinol, *n* = 5) group; and three treatment groups that were orally administered SH-P-1-1 at 200 (SH-L), 400 (SH-M), or 800 (SH-H) mg/kg (*n* = 5 per group) once daily for 21 d. The rats were euthanized after the experiment to collect tissue and blood samples. The levels of UA, creatinine (CRE), xanthine oxidase, adenosine deaminase (ADA) in the serum, and inflammatory factors tumor necrosis factor-*α* (TNF-α), interleukin-1β (IL-1β), and IL-6 in the kidney were determined using test kits (Jiancheng, China) in accordance with the manufacturer’s instructions. Posterior leg bones and kidneys were immersed in 4% paraformaldehyde (Aladdin, China), embedded in paraffin, and cut into 5 μm thick sections, which were then stained with hematoxylin and eosin (H&E). The severity of joint and kidney injuries was assessed and scored using a semi-quantitative analysis in Image J (version 1.8.0).

### 2.9 Gut microbiota analysis

The gut microbes were analyzed using the NGS Illumina high-throughput sequencing platform. Microbial DNA was isolated from fecal samples using the E.Z.N.A.^®^ soil DNA kit (Omega Bio-tek, USA). The 16S rDNA V3-V4 variable region was amplified using the 338F (5′-ACTCCTACGGGAGGCAGCA-3′)/806R (5′-GGACTACHVGGGTWTCTAAT-3′) primers. Quality control of fastq data was performed with fastp (version v0.12.4). The dada2 plugin of qiime2 (version 2020.2.0) was used to merge, remove duplicates, and filter chimeras of reads to generate representative sequences. Libraries were constructed with NEXTflex™ Rapid DNA-Seq Kit (Bioo Scientific, USA) and sequenced on a NovaSeq PE250 platform (Illumina, USA). Clustering of operational taxonomic units was performed based on 97% similarity of sequences. The UpSet analysis was used to illustrate the common and unique features among the different samples. Alpha-diversity was evaluated for gut microbial richness and diversity within the community, whereas beta-diversity was determined using principal coordinate analysis (PCoA) based on Jaccard and Bray–Curtis distances to assess microbial community separation and diversity among the samples. The gut microbiota with significant differences and associated categories among the groups were analyzed via linear discriminant analysis effect size (LEfSe). Random forest was performed to identify key microbes and metabolites associated with phenotypic changes.

### 2.10 Untargeted metabolomics analysis

Fecal specimens (50 mg) were suspended in 200 μL ultrapure water. To the suspension, 800 μL of a methanol–acetonitrile mixture (1:1, v/v) was added and the solution was ultrasonicated for 30 min. Proteins were then precipitated by incubating the extract at −20 °C for 1 h. The supernatant obtained after centrifugation (12,000 × g, 10 min, 4 °C) was dried under vacuum. The dry extract was dissolved in 200 μL of 30% acetonitrile, and the solution was centrifuged (12,000 × g, 4 °C) for 15 min. The supernatant, thus obtained, was analyzed via ultra-performance liquid chromatography (Vanquish, UPLC, Thermo, USA) and high-resolution mass spectrometry (QExactive HFX, Thermo, USA). Data were collected in ESI-positive (POS) or ESI-negative (NEG) mode. The original data were subjected to preprocessing, such as baseline filtering, peak identification, peak matching, retention time correction, and peak alignment using Progenesis QI (Waters Corporation, Milford, USA), and a data matrix was obtained containing retention time, mass-to-charge ratio, and peak intensity. Instrumental stability and reliability were evaluated by running quality control (QC) samples. The relative standard deviation of QC was utilized to reflect the quality of sequencing data. Inter- and intra-group differences in samples were analyzed using principal component analysis (PCA), whereas differences in metabolic profiles in each sample and between-group differential metabolites were examined using orthogonal projections to latent structures-discriminant analysis (OPLS-DA). The variable importance in projection (VIP) scores were used to represent the contribution of metabolites to differences in the OPLS-DA model, and *p*-values determined using the Student’s t-test indicated the significance of differences in metabolite levels. Fold change (FC) stated the ratio of the mean quantitative values of metabolites in the comparison groups. Differential metabolites were identified based on the following criteria: VIP > 1, *p* < 0.05, and FC > 1.5. A volcano plot was used to display metabolites with large variations and statistical significance. Hierarchical clustering was performed to analyze metabolite expression patterns in different samples, and Spearman’s heatmap, Procrustes analysis, canonical correspondence analysis (CCA), and Mantel test were used to visualize the differences in metabolite content between groups. The Kyoto encyclopedia of genes and genomes (KEGG) database was utilized for pathway enrichment to identify metabolic pathways in which differential metabolites were involved. Pearson’s correlation analysis was performed to evaluate the degree of correlation among different indicators.

### 2.11 Determination of SCFAs content

Fecal samples (50 mg) were dissolved in 30% phosphoric acid (50 μL, Sinopharm, China) and acetone (300 μL, ANPEL, China) by thorough mixing. The resultant mixture was centrifuged (12,000 × *g*, 4 °C) for 10 min and the supernatant, thus obtained, was analyzed using gas chromatography (Agilent 7,820, Agilent Technologies, USA) and quadrupole mass spectrometry (Agilent 5,977, Agilent Technologies, USA). The standard curve was fitted using the Quant-My-Way (Agilent Technologies, USA), and samples were absolutely quantified using an external standard approach.

### 2.12 Statistical analysis

Data were represented by mean ± standard deviation. A Student’s *t*-test was performed to assess differences between two groups, and a one-way analysis of variance with Dunnett’s multiple comparison test was used to evaluate significances among different groups using GraphPad Prism (version 8.0.2). A *p*-value < 0.05 was considered for statistical significance.

## 3 Results

### 3.1 Separation and purification of polysaccharides from *Phellinus igniarius*

**Figure 1:**
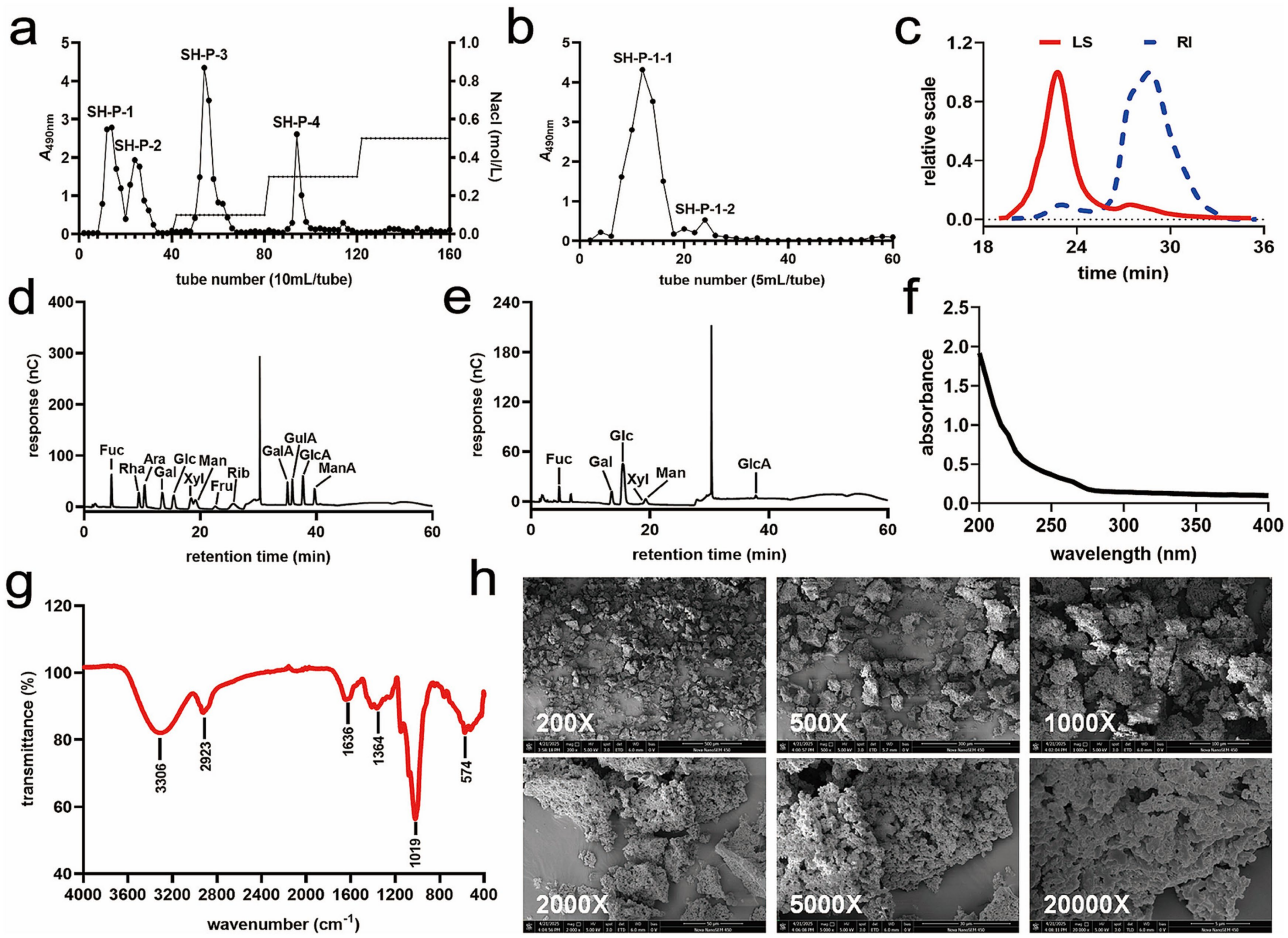
Purification of polysaccharides from *Phellinus igniarius* and their characterization. **(a)** Elution curve for *P. igniarius* polysaccharides chromatographed on DEAE-52 column; **(b)** Elution curve for Sephadex G-100 column; **(c)** Molecular weight of SH-P-1-1; **(d)** Monosaccharide standard; **(e)** Monosaccharide composition of SH-P-1-1; **(f)** Ultraviolet–visible (UV) spectrum of SH-P-1-1; **(g)** Fourier transform infrared (FT-IR) spectrum for SH-P-1-1; **(h)** Scanning electron microscopy (SEM) of SH-P-1-1.

Hot water extraction, a green and facile method for polysaccharides, was performed to extract the crude polysaccharides SH-Ps from *P. igniarius*. Four elution fractions, namely SH-P-1, SH-P-2, SH-P-3, and SH-P-4, were obtained by separating and purifying the crude SH-Ps using a DEAE-52 column (Figure 1a). Although the yield of SH-P-1 was not the highest, its inhibitory effect on the XOD activity *in vitro* was better than that of the other compounds (Supplementary Figure S1). Thus, collection and purification of SH-P-1 was performed using the Sephadex G-100 column. SH-P-1 was separated into two fractions based on its elution profile, and the main fraction, SH-P-1-1, was collected and freeze-dried for further analysis (Figure 1b).

### 3.2 Molecular weight and monosaccharide composition of SH-P-1-1

In the GPC-RI-MALS analysis, a single symmetrical peak was detected in both laser scattering (LS) signal and refractive index (RI) profiles of SH-P-1-1, suggesting that SH-P-1-1 was a homogenous and pure polysaccharide (Figure 1c). The weight average molecular weight (Mw) and the number average molecular weight (Mn) of SH-P-1-1 were 29.19 kDa and 23.04 kDa, respectively, with a polydispersity (Mw/Mn) of 1.27. This implied that the separation of SH-P-1-1 was very desirable. Monosaccharide composition analysis revealed that SH-P-1-1 comprised fucose (Fuc), galactose (Gal), glucuronic acid (GlcA), glucose (Glc), xylose (Xyl), and mannose (Man) in a molar ratio of 7.90:12.52:2.11:65.67:1.10:10.70 (Figures 1d,e).

### 3.3 UV–vis spectra and FT-IR spectroscopy

UV absorption peaks were not detected at 260 or 280 nm for SH-P-1-1, indicating the absence of proteins or nucleic acids in the polysaccharide preparations (Figure 1f). As evident from the FT-IR spectroscopy results, typical absorption peaks of SH-P-1-1 were consistent with those of polysaccharides within the 4,000–400 cm^−1^ range (28). Besides, a wide peak detected at 3306 cm^−1^ could be associated with O-H stretching vibrations (29), whereas a weak absorption peak at 2923 cm^−1^ was associated with the C-H stretching vibration (30). Furthermore, peaks detected around 1,636 cm^−1^ were linked to C=O stretching vibrations (31), that at 1364 cm^−1^ indicated C-O stretching vibrations (32), and that at 1019 cm^−1^, within 1,000–1,200 cm^−1^, was associated with C–O–C and C–O–H stretching vibrations (33). Additionally, the peaks at 574 cm^−1^ were associated with the pyranose ring (34) (Figure 1g). Overall, these results indicated the typical polysaccharide absorption characteristics of SH-P-1-1.

### 3.4 SEM analysis

SEM images of SH-P-1-1 at different magnifications are illustrated in Figure 1h. SH-P-1-1 exhibited an irregular block shape with a rough surface at 200 × to 1,000 × magnifications, respectively. The loose, porous structure was clearly observed on the surface of SH-P-1-1 at 2000 × to 20,000 × magnifications, respectively.

### 3.5 Methylation analysis

Linkage patterns of the glycosidic bonds in SH-P-1-1 were examined via gas chromatography-mass spectrometer analyses. SH-P-1-1 was predicted to consist of 14 glycosidic linkages, such as Fuc*p*-(1→, Man*p*-(1→, Glc*p*-(1→, →2) Fuc*p*-(1→, →3)-Man*p*-(1→, →2)-Man*p*-(1→, →2)-Glc*p*-(1→, →6)-Glc*p*-(1→,→4)-Glc*p*-(1→, →6)-Gal*p*-(1→,→3,4)-Glc*p*-(1→, →3,6)-Gal*p*-(1→, →4,6)-Glc*p*-(1→, →2,6)-Gal*p*-(1→, and the relative molar ratios were 1.02:2.92:25.18:2.0:5.07:3.37:1.35:3.37: 37.08:7.39:1.15:2.03:5.06:3.01 (Table 1). The ratios of glycosidic bonds derived from the methylation analysis conformed to the results of monosaccharide composition analysis.

**Table 1:** Linkage patterns of glycosidic bonds in SH-P-1-1 determined via gas chromatography–mass spectrometry.

Retention time (min)	Mass fragments (m/z)	Linkage type	Molar ratio (%)
7.502	72, 89, 102, 115, 118, 131, 162, 175	Fuc*p*-(1→	1.02
9.445	87, 102, 118, 129, 145, 161, 162, 205	Man*p*-(1→	2.92
9.521	87, 102, 118, 129, 145, 161, 162, 205	Glc*p*-(1→	25.18
11.033	89, 100, 115, 130, 131, 175, 190, 234	→2) Fuc*p*-(1→	2.00
12.867	87, 101, 118, 129, 161, 202, 234	→3)-Man*p*-(1→	5.07
13.052	88, 101, 129, 130, 161, 190, 205	→2)-Man*p*-(1→	3.37
13.182	88, 101, 129, 130, 161, 190, 205	→2)-Glc*p*-(1→	1.35
14.461	87, 99, 102, 118, 129, 162, 189, 233	→6)-Glc*p*-(1→	3.37
14.804	87, 102, 113, 118, 129, 162, 233	→4)-Glc*p*-(1→	37.08
16.248	87, 99, 102, 118, 129, 162, 189, 233	→6)-Gal*p*-(1→	7.39
16.993	87, 118, 129, 143, 185, 203, 305	→3,4)-Glc*p*-(1→	1.15
18.533	87, 101, 118, 129, 160, 189, 234	→3,6)-Gal*p*-(1→	2.03
19.101	85, 102, 118, 127, 159, 162, 201, 261	→4,6)-Glc*p*-(1→	5.06
20.442	87, 88, 99, 100, 129, 130, 189, 190	→2,6)-Gal*p*-(1→	3.01

### 3.6 Effects of SH-P-1-1 on serum biochemical parameters and tissue injury in rats

**Figure 2:**
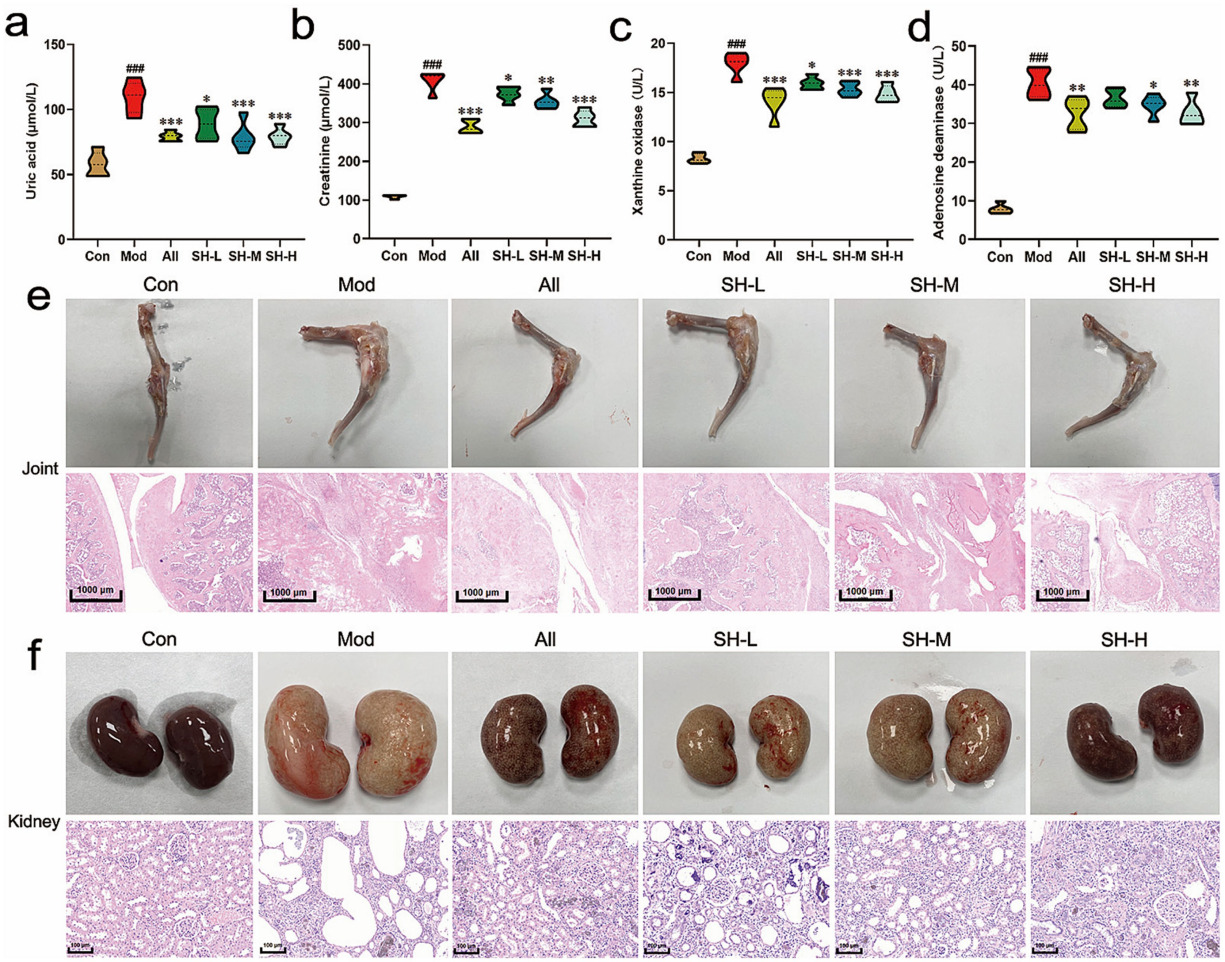
Effects of SH-P-1-1 on hyperurecemia (HUA) and gouty arthritis in rats. **(a)** Uric acid (UA); **(b)** Creatinine (CRE); **(c)** Xanthine oxidase (XOD); **(d)** Adenosine deaminase (ADA); **(e)** Femur joint swelling and hematoxylin and eosin (H&E)-stained sections; scale bar = 1,000 μm; **(f)** Morphological changes in the kidney and images of H&E-stained sections; scale bar = 100 μm. ###, *p* < 0.001 vs. Con; *, *p* < 0.05 vs. Mod; **, *p* < 0.01 vs. Mod; ***, *p* < 0.001 vs. Mod. *n* = 5.

The levels of UA (109.33 ± 12.0 μmol/L vs. 57.78 ± 9.43 μmol/L, *p* < 0.001) and CRE (406.98 ± 26.0 μmol/L vs. 108.28 ± 4.41 μmol/L, *p* < 0.001), and the activities of XOD (17.94 ± 1.15 U/L vs. 8.28 ± 0.46 U/L, *p* < 0.001) and ADA (17.94 ± 1.15 U/L vs. 8.28 ± 0.46 U/L, *p* < 0.001) in the serum of rats were remarkably elevated in the Mod group compared with those in the Con group. SH-P-1-1 treatment significantly and dose-dependently lowered the serum UA and CRE levels. It reduced the activities of XOD and ADA by 27.64% (*p* < 0.001), 23.78% (*p* < 0.001), 17.11% (*p* < 0.001), and 18.75% (*p* < 0.01) at a dose of 800 mg/kg, relative to the values in the Mod group (Figures 2a–d). The renal inflammatory cytokines TNF-*α* (*p* < 0.001), IL-6 (*p* < 0.01), and IL-1β (*p* < 0.001) in the Mod group were significantly increased compared with the Con group. SH-P-1-1 treatment had a notable anti-inflammatory effect in a dose-dependent manner, as seen by the noteworthy reduction of TNF-α (*p* < 0.001), IL-6 (*p* < 0.01) and IL-1β (*p* < 0.01) at a dose of 800 mg/kg (Supplementary Figures S2a-c). Additionally, rat joints in the model group showed severe damage; the degree of swelling and cavity space was significantly increased, and inflammatory cell infiltration occurred in the synovial tissue. SH-P-1-1 supplementation significantly ameliorated joint swelling, decreased inflammatory cell infiltration and synovial cell proliferation in rats (Figure 2e; Supplementary Figure S2d). Moreover, rat kidneys in the model group showed obvious enlargement; the kidneys were white, with markedly elevated indices compared with those in the Con group. However, the degree of kidney swelling in rats treated with different doses of SH-P-1-1 was significantly improved, and the kidney indices were significantly decreased; however, no obvious changes were observed in the color of the kidneys compared with that in the model rats. H&E staining revealed that HUA and gout caused severe kidney damage as indicated by tubule dilation, glomerular atrophy, inflammatory cell infiltration within the renal interstitium, and epithelial cell swelling. SH-P-1-1 treatment significantly improved the degree of glomerular lesions and alleviated renal tubular injury and inflammatory cell infiltration (Figure 2f; Supplementary Figure S2e).

### 3.7 Function of SH-P-1-1 in regulating the gut microbiota composition in rats

**Figure 3:**
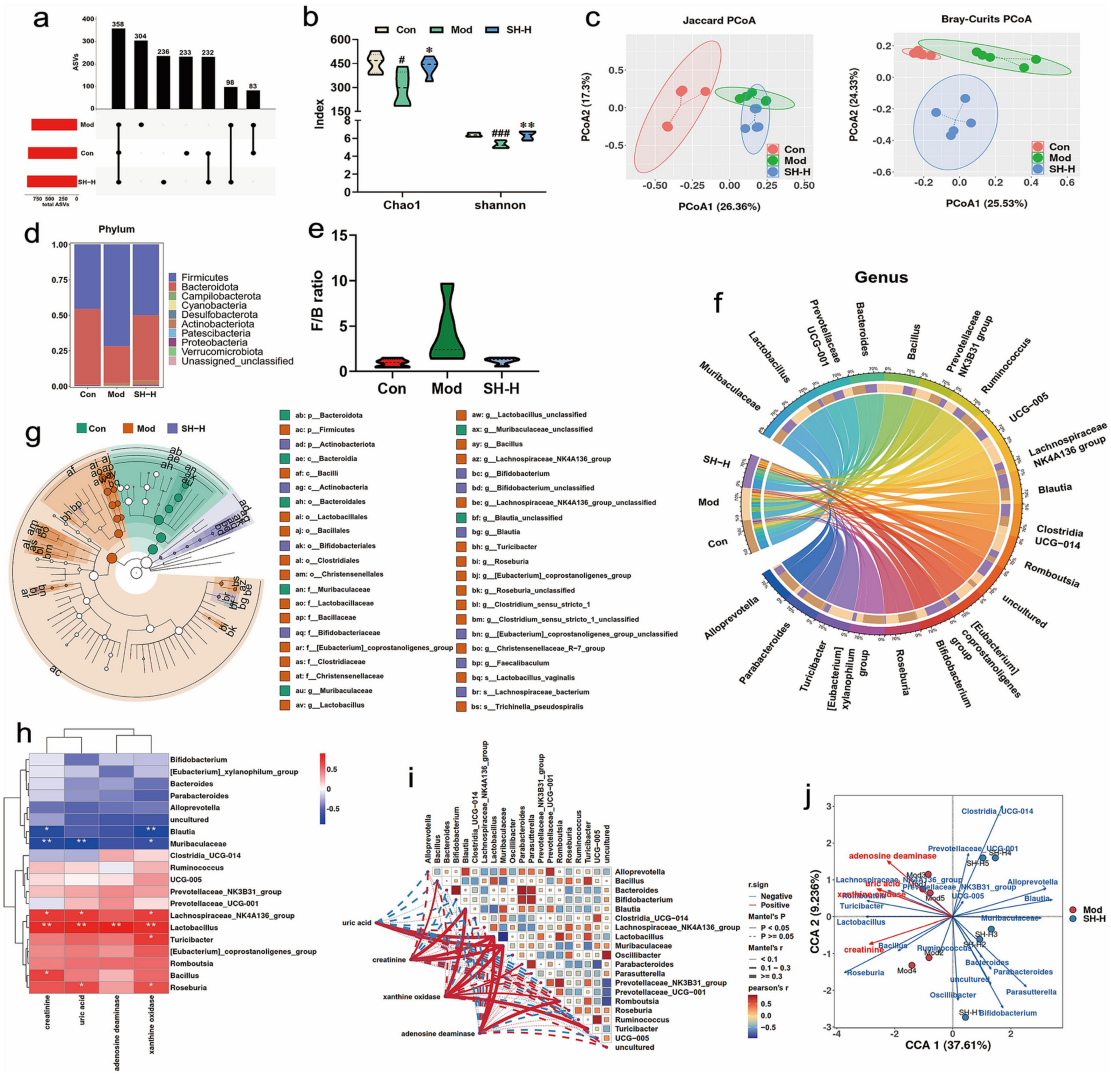
Regulation of SH-P-1-1 on the gut microbial composition and structure. **(a)** Venn analysis for the gut microbiota according to amplicon sequence variant (ASV) features; **(b)** Alpha-diversity analyses based on Chao1 and Shannon indices; **(c)** Beta-diversity analyses according to Bray–Curtis and Jaccard principal coordinate analysis (PCoA); **(d)** Gut microbial composition profile at the phylum level; **(e)** *Firmicutes*/*Bacteroidetes* (F/B) ratio; **(f)** Relative gut microbial abundance profile at the genus level; **(g)** Identification of key specific bacterial taxa via the linear discriminant analysis effect size (LEfSe) analysis; **(h–j)** Relation of uric acid (UA), creatinine (CRE), xanthine oxidase (XOD), adenosine deaminase (ADA) and the intestinal flora. #, *p* < 0.05 vs. Con; ###, *p* < 0.001 vs. Con; *, *p* < 0.05 vs. Mod; **, *p* < 0.01 vs. Mod. *n* = 5.

A total of 358 common features were identified among the three groups, occupying approximately 23.19% of the overall amplicon sequence variants (ASVs), and 233, 304, and 236 unique ASVs were detected in the control, gout, and SH-H groups, respectively (Figure 3a). Consequently, the gut microbial composition changed significantly in the three groups. The Mod group had markedly lower Chao1 (293.83 ± 106.19 vs. 451.55 ± 61.39, *p* < 0.05) and Shannon (5.30 ± 0.39 vs. 6.39 ± 0.18, *p* < 0.001) indices than the Con group. However, SH-P-1-1 significant reversed the Chao1 (428.58 ± vs. 293.83 ± 106.19, *p* < 0.05) and Shannon (6.34 ± 0.44 vs. 5.30 ± 0.39, *p* < 0.01) indices compared with those in the Mod group, indicating the notably increased community diversity in the SH-H group relative to that in the Mod group (Figure 3b). From Jaccard and Bray–Curtis distance-based PCoA, data within groups exhibited clustering, whereas a separation was observed between the groups, revealing a distinct gut microbial composition among the three groups (Figure 3c). At phylum to genus levels, relative gut microbial abundance in each group was calculated to analyze the dominant components. Firmicutes and Bacteroidota were the main gut microbial components in each group at the phylum level (Figure 3d). Relative to that in the Con group, the abundance of Firmicutes increased and that of Bacteroidota decreased, with an increased Firmicutes/ Bacteroidota (F/B) ratio in the Mod group. However, SH-H reduced the F/B ratio relative to that in the Mod group (Figure 3e). At the genus level, the abundance of *Bacillus* (*p* < 0.01), *Lachnospiraceae_NK4A136_group* (*p* < 0.05), *Romboutsia* (*p* < 0.05), and *Turicibacter* (*p* < 0.01) was remarkably increased, whereas that of *Muribaculaceae* (*p* < 0.001) was markedly decreased in the Mod group compared with that in the Con groups. Nonetheless, SH-P-1-1 supplementation dramatically increased the abundance of *Blautia* (*p* < 0.05) and *Muribaculaceae* (*p* < 0.001) but decreased that of *Lachnospiraceae_NK4A136_group* (*p* < 0.05), *Lactobacillus* (*p* < 0.001), and *Turicibacter* (*p* < 0.01) in the SH-H group relative to that in the Mod group (Figure 3f; Supplementary Figure S3). Supplementary Figure S4 shows the classification of the intestinal flora at class, order, family, and species levels. Critical bacterial taxa with differences between two groups were identified via the LEfSe analysis. Actinobacteriota (phylum), Actinobacteria (class), Bifidobacteriales (order), Bifidobacteriaceae (family), *Bifidobacterium* (genus), *Bifidobacterium*_*unclassified* (genus), *Blautia* (genus), *[Eubacterium]_coprostanoligenes_group_unclassified* (genus), *Faecalibaculum* (genus), and *Lachnospiraceae*_*bacterium* (species) were the dominant bacterial taxa in the SH-H group (Figure 3g). Notably, from the phylum to the genus level (Actinobacteriota, Actinobacteria, Bifidobacteriales, Bifidobacteriaceae, and *Bifidobacterium*), *Bifidobacterium* was the dominant bacterial taxon in the SH-H group, indicating that it may be the main target microorganism related to the regulatory activity of SH-P-1-1. As indicated by the correlation analysis, UA levels showed an obviously positive correlation with *Lachnospiraceae_NK4A136_group* (*p* < 0.05), *Lactobacillus* (*p* < 0.01), and *Roseburia* (*p* < 0.05) but a negative correlation with *Muribaculaceae* (*p* < 0.01). CRE levels displayed a positive correlation with *Lachnospiraceae_NK4A136_group* (*p* < 0.05), *Lactobacillus* (*p* < 0.01), and *Bacillus* (*p* < 0.05), and a negative correlation with *Muribaculaceae* (*p* < 0.01) and *Blautia* (*p* < 0.05). Moreover, the ADA activity showed a positive correlation with *Lactobacillus* (*p* < 0.01); XOD activity demonstrated a positive correlation with *Lachnospiraceae_NK4A136_group* (*p* < 0.05), *Lactobacillus* (*p* < 0.01), *Roseburia* (*p* < 0.05), *Turicibacter* (*p* < 0.05) and a negative correlation with *Muribaculaceae* (*p* < 0.05) and *Blautia* (*p* < 0.01) (Figures 3h–j). Collectively, SH-P-1-1 improved HUA and gout via modulating gut microbial composition and structure.

### 3.8 SH-P-1-1 enhanced the rat fecal metabolomic profile

**Figure 4:**
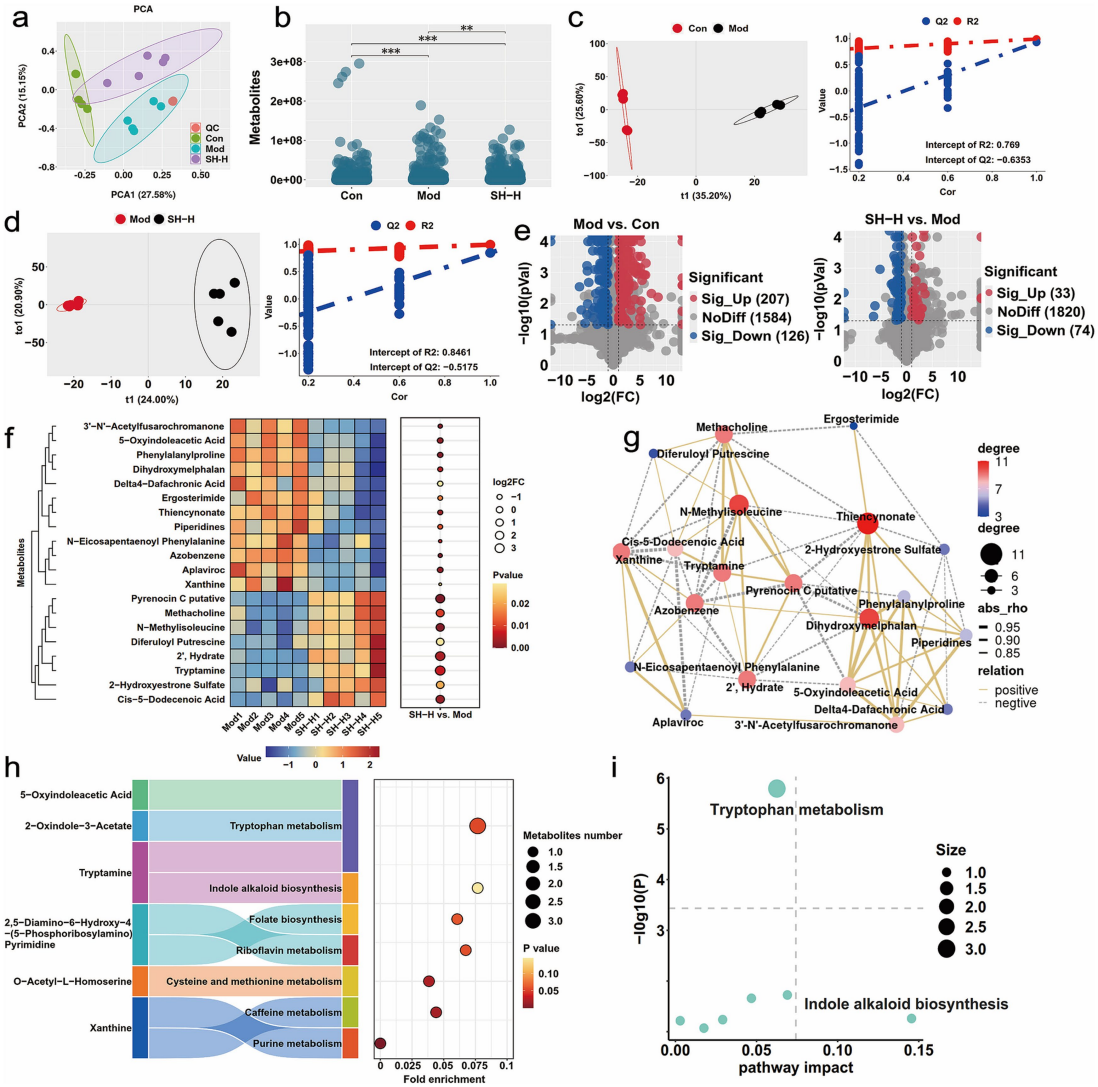
Untargeted metabolomic analysis in the positive (POS) mode. **(a)** Differences and variation analysis for the samples via principal components analysis (PCA); **(b)** Differential analysis of metabolites among the different groups; **(c)** Feature analysis of differential metabolites in the Con versus Mod group comparisom via the orthogonal projections to latent structures-discriminant analysis (OPLS-DA) and permutation testing; **(d)** Feature analysis of differential metabolites between the Mod and SH-H via the OPLS-DA and permutation testing; **(e)** Differential metabolites in the Con versus Mod and Mod versus SH-H group comparisons displayed via a volcano plot; **(f)** Hierarchical clustering analysis for differential metabolites in the Mod versus SH-H group comparison (top 20); **(g)** Correlation analysis for differential metabolites; **(h)** Key pathways associated with differential metabolites; **(i)** Kyoto encyclopedia of genes and genomes (KEGG) enrichment for differential metabolites. **, *p* < 0.01; ***, *p* < 0.001. *n* = 5.

PCA was conducted for determining total intra- and inter-sample differences. Data were clearly separated among groups and clustered within each group (Figure 4a; Supplementary Figure S5a), and significant differences could be detected in two groups (Figure 4b; Supplementary Figure S5b). Consequently, SH-P-1-1 had a significant effect on the fecal metabolic profile of rats. Moreover, significant differences were observed in these comparisons on OPLS-DA score plots, with all samples within the 95% confidence interval. Based on permutation tests, these models were robust, with no overfitting (Figures 4c,d; Supplementary Figures S5c,d). A total of 333 significant differential metabolites, including 207 upregulated and 126 downregulated metabolites, were identified in the Mod versus Con group comparison, and 107 significant differential metabolites, including 33 upregulated and 74 downregulated metabolites, were identified in the SH-H versus Mod group comparison in the POS mode (Figure 4e). Significant differential metabolites in the NEG mode are shown in Supplementary Figure S5e. Hierarchical clustering analysis revealed that the levels of pyrenocin C putative, methacholine, *n*-methylisoleucine, diferuloyl putrescine, 2′, hydrate, tryptamine, 2-hydroxyestrone sulfate, and *cis*-5-dodecenoic acid were markedly elevated in the POS mode (Figure 4f; Supplementary Figure S6), and those of n-palmitoyl phenylalanine, biotin, isopentenyladenine, pteroside B, and ethylcholine mustard were significantly increased in the NEG mode in the SH-H group compared with those in the Mod group (Supplementary Figure S7a). The increased or decreased abundance of metabolites showed an obvious positive relation (Figure 4g; Supplementary Figure S7b).

The KEGG enrichment was carried out for differential metabolite-related metabolic pathways, which mainly included tryptophan metabolism, purine metabolism, caffeine metabolism, riboflavin metabolism, cysteine and methionine metabolism, indole alkaloid biosynthesis, and folate biosynthesis in the POS mode in the SH-H vs. Mod group comparison (Figure 4h). The KEGG pathway topology showed that tryptophan metabolism was the most significantly correlated with the changes of differential metabolites (Figure 4i). SH-P-1-1 intervention reversed the decreasing trend for tryptophan-derived metabolites to varying degrees (Supplementary Figure S8). Moreover, the levels of indole (*p* < 0.05) and kynurenine (*p* < 0.05), especially tryptamine (*p* < 0.01) in the SH-H group significantly increased compared with that in the Mod group (Supplementary Figures S6f, S8a-b). The results of the KEGG enrichment and KEGG pathway topology analyses for differential metabolites in NEG mode are illustrated in Supplementary Figure S9.

**Figure 5:**
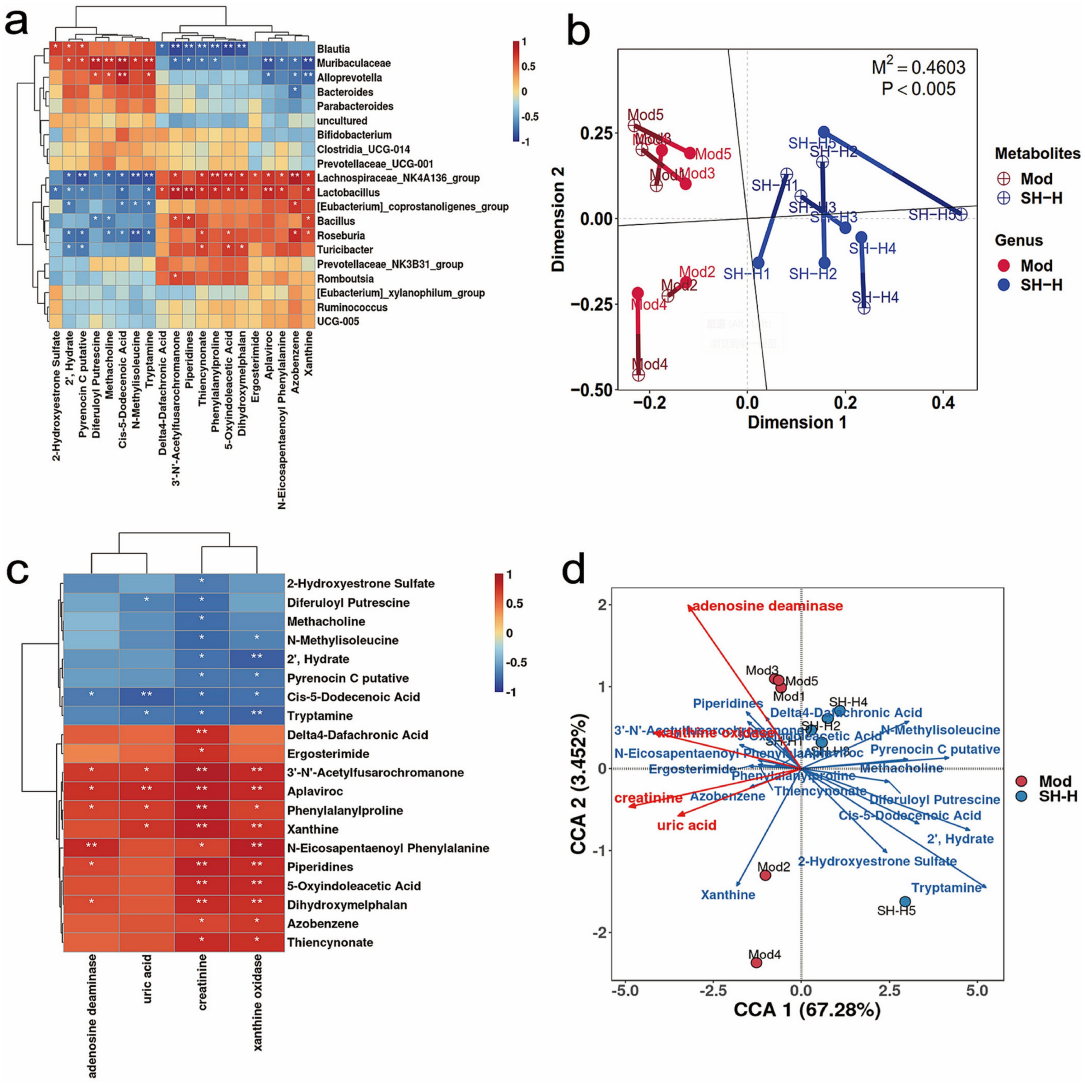
Correlation of the abundance of intestinal microbiota with differential metabolites in positive (POS) mode between the Mod and SH-H groups. **(a,b)** Hierarchical clustering and procrustes analysis for the relation of the differential metabolites and gut microbiota; **(c,d)** Correlation among uric acid (UA), creatinine (CRE), xanthine oxidase (XOD), adenosine deaminase (ADA) and the differential metabolites. *, *p* < 0.05; **, *p* < 0.01. *n* = 5.

Correlation analysis was conducted for the intestinal microbiota and differential metabolites to explore the mechanisms by which SH-P-1-1 alleviates HUA and gout. The gut microbes, such as *Blautia* and *Muribaculaceae*, were significantly positively correlated with metabolites markedly elevated in the SH-H group. However, *Lachnospiraceae_NK4A136_group*, *Lactobacillus*, and *Turicibacter*, the markedly decreased groups in the SH-H group, showed a significant and negative association with these metabolites (Figure 5a; Supplementary Figure S10a). Procrustes analysis also showed a strong synergy between gut microbiota and fecal metabolic profiles (Figure 5b). Moreover, UA and CRE levels and XOD and ADA activities were also significantly negatively related to the metabolites that were markedly elevated in the SH-H group and positively associated with those that were dramatically decreased in the SH-H group (Figures 5c,d; Supplementary Figure S10b). Therefore, SH-P-1-1 is likely to increase the abundance of *Muribaculaceae* or reduce that of *Lachnospiraceae_NK4A136_group* and *Lactobacillus*, thereby promoting the production of differential metabolites, such as tryptophan-derived metabolites, which are closely related to tryptophan metabolism to synergistically maintain health.

### 3.9 Microbial and metabolites biomarkers identification

**Figure 6:**
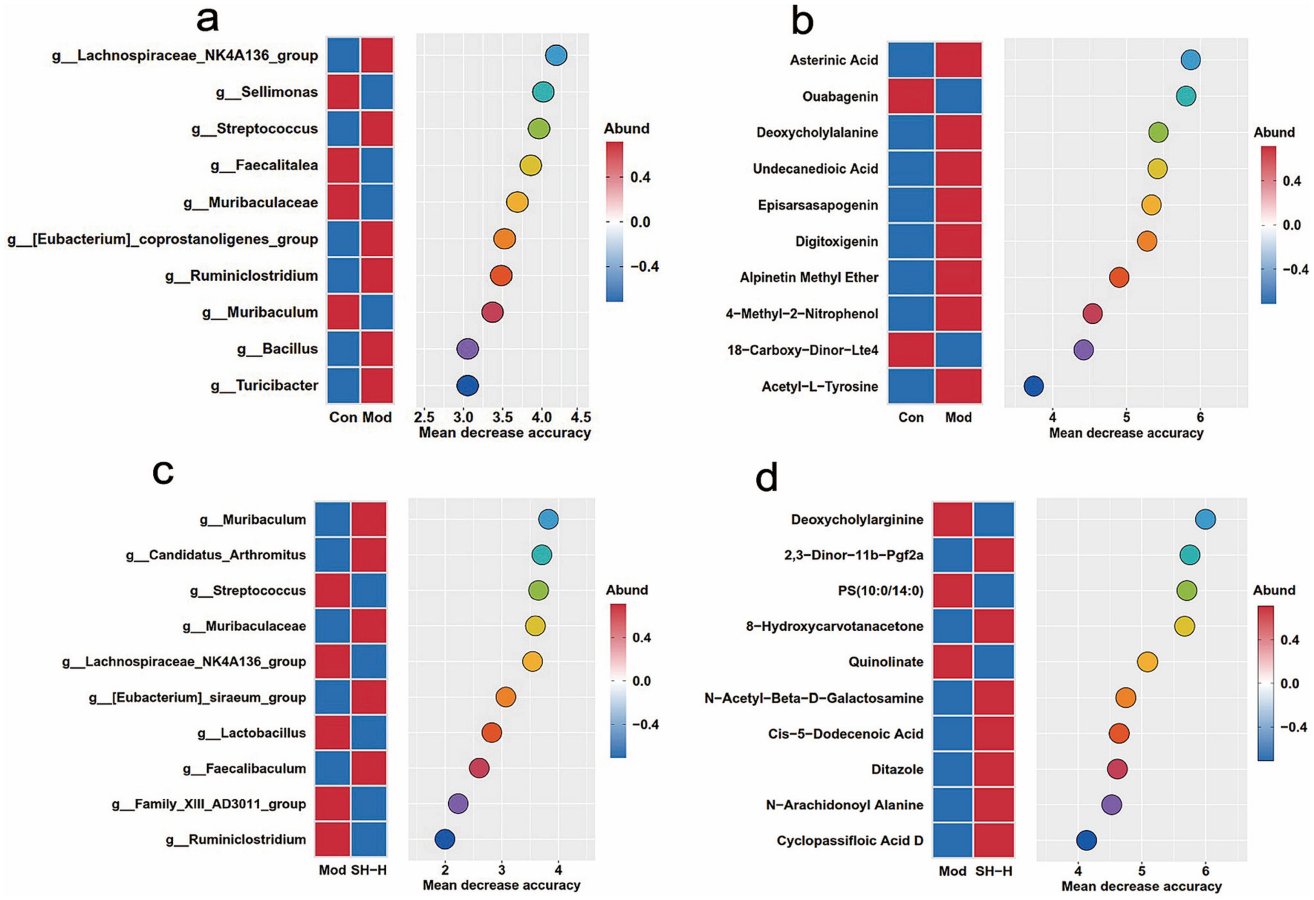
Gut microbiota and metabolomic markers of the rats in Mod **(a,b)** and SH-H **(c,d)** were identified using random forests analysis (Top 10). *n* = 5.

The random forest analysis was performed to investigate whether gut microbial and metabolomic parameters could serve as markers for the diagnosis and treatment of HUA and gout. The results showed that *Lachnospiraceae_NK4A136_group* was a highly labeled microbiota, and the metabolite was asterinic acid for determining HUA and gout in rats (Figures 6a,b). Furthermore, *Muribaculum* and deoxycholylarginine were the major microbiota and metabolite features in rats after SH-P-1-1 treatment (Figures 6c,d). These outcomes suggest that the diagnosis and treatment of HUA and gout, driven by specific gut microbiota as well as metabolites are a viable strategy.

### 3.10 Effects of SH-P-1-1 on SCFA profiles in rats

**Figure 7:**
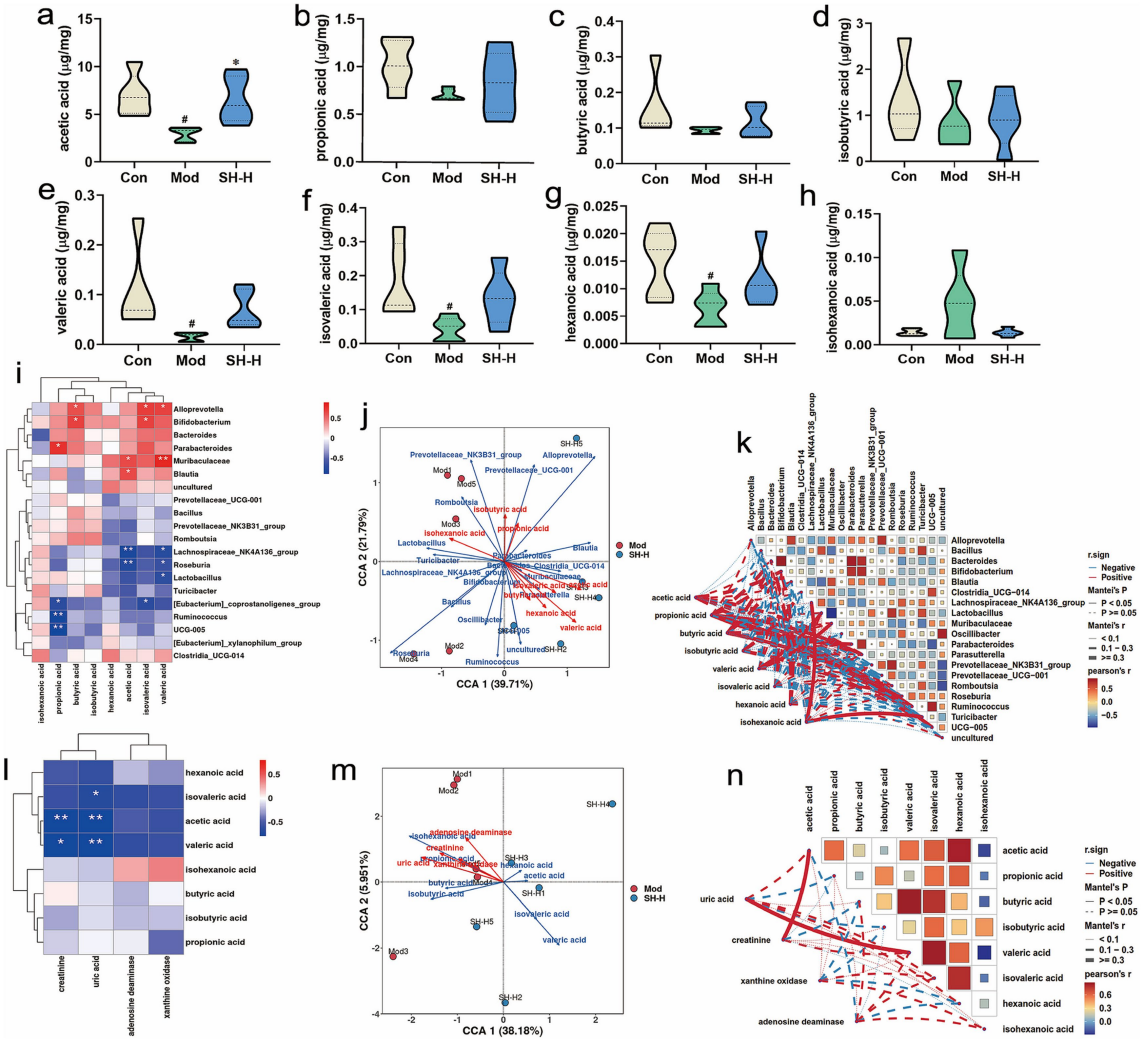
Short-chain fatty acid (SCFA) profiles after SH-P-1-1 intervention. **(a–h)** Levels of acetic, propionic, butyric, isobutyric, valeric, isovaleric, hexanoic, and isohexanoic acids. #, *p* < 0.05 vs. Con; *, *p* < 0.05 vs. Mod; **(i–k)** Spearman’s heatmap, canonical correspondence analysis (CCA) and Mantel analysis for the correlation of the gut microbiota with SCFAs; **(l-n)** Hierarchical clustering, CCA and Mantel analysis for the correlation among uric acid (UA), creatinine (CRE), xanthine oxidase (XOD), adenosine deaminase (ADA), and SCFAs. *, *p* < 0.05; **, *p* < 0.01. *n* = 5.

SCFAs, which are important metabolites of the intestinal microbiota, are relevant signaling molecules for HUA and gout. Relative to that in the Con group, the content of acetic acid (2.92 ± 0.73 μg/mg vs. 6.99 ± 2.22 μg/mg, *p* < 0.05), valeric acid (0.015 ± 0.0088 μg/mg vs. 0.11 ± 0.085 μg/mg, *p* < 0.05), isovaleric acid (0.043 ± 0.035 μg/mg vs. 0.18 ± 0.11 μg/mg, *p* < 0.05), and hexanoic acid (0.064 ± 0.0033 μg/mg vs. 0.015 ± 0.0061 μg/mg, *p* < 0.05) declined considerably in the Mod group. This indicated that HUA and gout dramatically decreased the content of SCFAs in the rat gut, likely associated with gut microbial dysbiosis. However, SH-P-1-1 intervention reversed the decreasing trend for SCFA levels to varying degrees. Moreover, the level of acetic acid (6.52 ± 2.45 μg/mg vs. 2.92 ± 0.73 μg/mg, *p* < 0.05) in the SH-H group apparently increased compared with that in the Mod group (Figures 7a–h). Correlation analysis indicated that the SCFA levels were closely related to the intestinal microbiota. For example, acetic acid was significantly positively related to *Blautia* (*p* < 0.05) and *Muribaculaceae* (*p* < 0.05), and negatively related to *Lachnospiraceae_NK4A136_group* (*p* < 0.01) and *Roseburia* (*p* < 0.01) (Figures 7i–k). Moreover, UA had a significant and negative relation with valeric acid (*p* < 0.01), acetic acid (*p* < 0.01), and isovaleric acid (*p* < 0.05), whereas CRE was significantly and negatively related to acetic acid (*p* < 0.01) as well as valeric acid (*p* < 0.05) (Figures 7l–n). These findings indicated that SH-P-1-1 regulates SCFA levels by modulating SCFA-producing gut microbial composition and abundance, which may also be one of the mechanisms by which SH-P-1-1 improves HUA and gout in rats.

## 4 Discussion

Natural polysaccharides show significant potential as safe and effective HUA and gout therapies (35). PRNP-1 and PRNP-2, the heteropolysaccharides extracted from blackcurrant, could significantly decrease the levels of UA, CRE, and the activity of XOD in the serum of hyperuricemic mice (36). A water-soluble polysaccharide obtained from *Lonicera japonica* decreased serum UA levels, suppressed XOD activity, and relieved gouty arthritis (37). These findings highlight the prospects of polysaccharides to improve HUA and gout, and could provide new strategies and methods for designing potential candidate drugs for these diseases. The present study explored the therapeutic activity of *P. igniarius* polysaccharide SH-P-1-1 in alleviating gout by establishing a rat model of HUA and acute gout inflammation. SH-P-1-1 significantly decreased the serum UA and CRE content and XOD and ADA activities in a dose-dependent manner. Moreover, SH-P-1-1 reduced kidney and joint swelling in rats. These results indicate that SH-P-1-1 may be a therapeutic agent for preventing and treating HUA and gout. However, the underlying mechanisms require further elucidation.

The functional characteristics of polysaccharides are tightly related to their structures (38). Molecular weight, glycosidic linkage type, and monosaccharide composition are strongly correlated with the bioactivity and physical properties of polysaccharides (24, 39, 40). Han et al. explored the effects of alginate with different molecular weights in hyperuricemic mice. Low-molecular-weight alginate improved bioavailability via microbial biodegradation and increased therapeutic efficacy against HUA (41). Wang et al. separated acidic and neutral polysaccharides (ACSP and NCSP, respectively) from corn silk. NCSP includes Man, GlcA, Glc, Gal, and Ara, whereas ACSP contains galactose acid (GalA). The molecular weight of NCSP was lower than that of ACSP, but NCSP exhibited higher activity against HUA, significantly improved kidney damage, and promoted UC excretion (42). Polysaccharides with (1 → 3), (1 → 4), or (1 → 6) glycosidic bonds in their main chain are effective in modulating gut health (43, 44). SH-P-1-1 was characterized as a homogenous, low-molecular-weight (29.16 kDa) polysaccharide and included fucose, galactose, glucose, xylose, mannose, and glucuronic acid. Moreover, we identified the glycosidic residues present in SH-P-1-1. These structural features indicate that SH-P-1-1 has good potential for improving HUA and gout.

Dysbiosis of the intestinal microbiota can affect UA metabolism, raising serum UA levels (25). The SCFA-producing bacteria, such as *Ruminococcus*, *Lachnospiraceae*, *Roseburia*, and *Bifidobacterium*, correlate negatively with UA levels (45). *Lactobacillus* decreases the adsorption of purine onto the intestine to attenuate the increase in serum UC levels and exacerbation of HUA (46). Therefore, specifically regulating the gut microbiota is a strategy for alleviating and treating HUA. Polysaccharides can alleviate and reverse HUA and gout by modulating the intestinal microbiota and the metabolites (24). Intervention with *Imperata cylindrica* polysaccharides markedly increased the abundance of *Lactobacillus* and *Bifidobacterium* and modulated the levels of metabolites, including SCFAs, bile acids, and indole, exerting a therapeutic effect in mice with hyperuricemic nephropathy (47). *Sporisorium reiliana* polysaccharide treatment significantly increased the abundance of *Muribaculum*, *Prevotella*, and *Muribaculum intestinale* and decreased the levels of uridine, hippuric acid, kynurenic acid, arachidonoyl, propionic acid, and metabolites related to inflammatory pathways to improve gout and kidney injury (25). In this study, SH-P-1-1 supplementation significantly enriched *Blautia* and *Muribaculaceae* relative to that in the Mod group. *Muribaculaceae* and *Blautia* are the SCFA-generating bacteria involved in improving inflammation and enhancing immunity (48–50). An elevated abundance of *Muribaculaceae and Blautia* could alleviate HUA and gout by enhancing intestinal propionate and butyrate concentration (49, 51). Ethanol extract of *Torreya grandis* seeds increased the abundance of *Muribaculaceae* and *Blautia* to enhance UA excretion and modulate oxidative stress and inflammation in the damaged tissues of HUA mice (52). Additionally, SH-P-1-1 increased the levels of metabolites, such as pyrenocin C putative, methacholine, and diferuloyl putrescine, compared with their respective levels in the Mod group. Moreover, SH-P-1-1 intervention increased the levels of SCFAs, especially acetic acid, which was markedly higher than in the Mod group. Pathways enriched for differential metabolites in the SH-H vs. Mod group comparison mainly included purine metabolism, tryptophan metabolism, caffeine metabolism, cysteine and methionine metabolism, riboflavin metabolism, indole alkaloid biosynthesis, and folate biosynthesis in the POS mode.

Tryptophan and purine metabolisms are closely correlated with HUA (53). Caffeine metabolism is involved in the degradation of UA (54). L-cysteine and L-methionine, which are involved in cysteine and methionine metabolism, are negatively correlated with the occurrence of gout (55). Differential metabolites involved in riboflavin metabolism can decrease UA levels and can be used to prevent and treat HUA (56). Folates and indoles, which are involved in folate and indole alkaloid biosynthesis, are negatively associated with the risk of gout (57, 58). Notably, KEGG pathway topology indicated that tryptophan metabolism showed the strongest correlation with the changes in differential metabolites. Tryptophan is recognized for its therapeutic potential in treating HUA due to its ability to regulate the uric acid metabolism (59). Gut microbes can also directly convert tryptophan into various secondary metabolites, such as tryptamine, indole and its derivatives, or kynurenine (60). Tryptamine, indole and its derivatives have been proven to regulate the composition of the intestinal microbiota, influence the growth of beneficial bacteria, and inhibit pathogenic bacteria (61). They could act as the aryl hydrocarbon receptor ligands, contribute to maintaining immune homeostasis and preserving the integrity of the intestinal barrier, and contribute to the alleviation of HUA and gout (62). Kynurenine represents an important microbiota-associated metabolite and biomarker of gut function (63). It can also bind to the aryl hydrocarbon receptors and activate kynurenine-dependent immune regulation, and reduce the degree of inflammation in gout (64). These results indicated that SH-P-1-1 regulates the gut microbiota and its metabolic profiles to improve HUA and gout.

Although this study indicated that SH-P-1-1 shows considerable potential for the treatment of HUA and gout, the limitations of the present research need to be highlighted. Firstly, this was only a preliminary research on the structure–activity relationship of SH-P-1-1, warranting further methodological investigation to determine its precise chemical structure. For example, nuclear magnetic resonance can be used to investigate the linkages and sequences of sugar residues in the polysaccharides (65). Moreover, new computational methods, such as molecular dynamics simulation and molecular docking, can be used to explore the structure–activity of polysaccharides (66). Therefore, traditional characterization techniques can be combined with computational methods to analyze the structure–activity relationship for SH-P-1-1. Secondly, the detailed mechanisms underlying the anti-gout effects of SH-P-1-1 were not fully explored and need further investigation. *Dioscorea septemloba* polysaccharides reportedly decreased serum UA levels, regulate the expression of urate transporter, and relieve kidney and liver damage by suppressing the LPS-TLR4-MYD88-NF-κB pathway in HUA mice (67). *I. cylindrica* polysaccharides effectively inhibited the intestinal NF-κB pathway, thereby suppressing inflammatory factor production while modulating gut homeostasis (47). Therefore, identifying the signaling pathways and key transporters and regulators targets of SH-P-1-1 is an important challenge for future research. Thirdly, although this study evaluated the correlation between gut microbiota and differential metabolites, the causal relationship has not been investigated through intervention methods. Hence, it is necessary to adopt fecal microbiota transplantation or screen specific metabolites to verify their regulatory relationships in the future. Finally, research on the anti-gout effects of polysaccharides has mainly focused on animal models, and there is a lack of clinical studies to verify whether polysaccharides are safe and effective in the human body. Moreover, studies regarding potential side effects induced by polysaccharide interventions, especially the risks related to treatment duration and dosage, remain unexplored. Hence, evaluating the efficacy of SH-P1-1 in treating gout in clinical trials and monitoring its potential toxicity and adverse effects are primary directions for future research.

## Data Availability

The original contributions presented in the study are publicly available. This data can be found at: https://www.ncbi.nlm.nih.gov/bioproject/PRJNA1375444.
