# Supplementary material for: Protective effects of polysaccharide SH-P-1-1 isolated from Phellinus igniarius on hyperuricemia and gout via intestinal microecosystem regulation
Source: Front Nutr. 2025 Dec 19;12:1717561. doi: 10.3389/fnut.2025.1717561 (PMC12757238; doi:10.3389/fnut.2025.1717561)
Supplement: Supplementary file 1 [file Table_1.DOCX]

Supplementary Material

**Protective effects of polysaccharide SH-P-1-1 isolated from *Phellinus igniarius* on hyperuricemia and gout via intestinal microecosystem regulation**

**Yanan Wang^1, 2 †^, Xinyi Qian^1 †^, Lingzhi Chen^3^, Ling Yang^1^, Zhenjiang Zhang^1^, Zhilong Qu^1^, Yuhan Yang^1^, Yihao Li^1^, Zaizhong Ni^1, 2*^, Ying Shao^1, 2^, Anhui Chen^1, 2 *^**

^1^College of Food and Bioengineering, Xuzhou University of Technology, Xuzhou, Jiangsu, China

^2^Jiangsu Key Construction Laboratory of Food Resource Development and Quality Safe, Xuzhou University of Technology, Xuzhou, Jiangsu, China

^3^School of Biotechnology, Jiangnan University, Wuxi, Jiangsu, China

*** Correspondence:**

Zaizhong Ni: [nizaizhong@163.com](mailto:nizaizhong@163.com)

Anhui Chen: [chenah201@163.com](mailto:chenah201@163.com)

† These authors have contributed equally to this work.

# Supplementary Data

Supplementary Material should be uploaded separately on submission. Please include any supplementary data, figures and/or tables.

Supplementary material is not typeset so please ensure that all information is clearly presented, the appropriate caption is included in the file and not in the manuscript, and that the style conforms to the rest of the article.

# Supplementary Figures and Tables

For more information on Supplementary Material and for details on the different file types accepted, please see [here](https://www.frontiersin.org/guidelines/author-guidelines#supplementary-material).

## Supplementary Figures


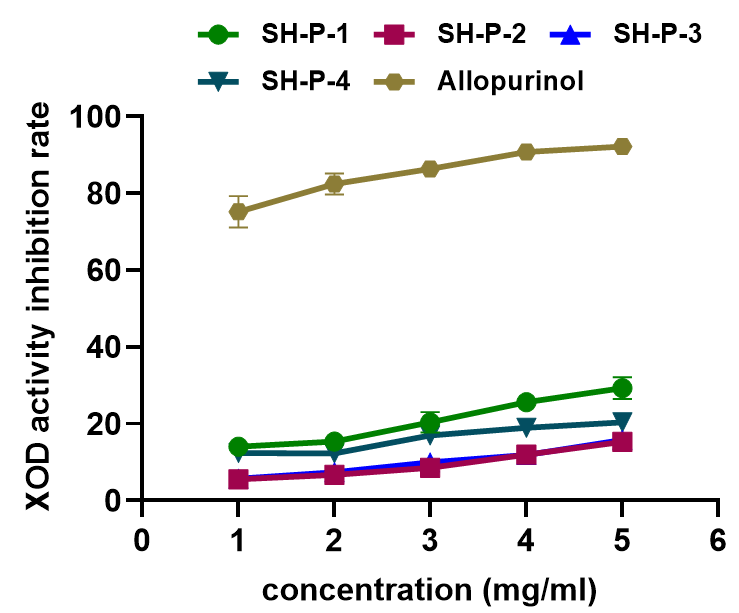


**Fig. S1** Inhibitory effect on the XOD activity of SH-P-1, SH-P-2, SH-P-3 and SH-P-4 *in vitro*.


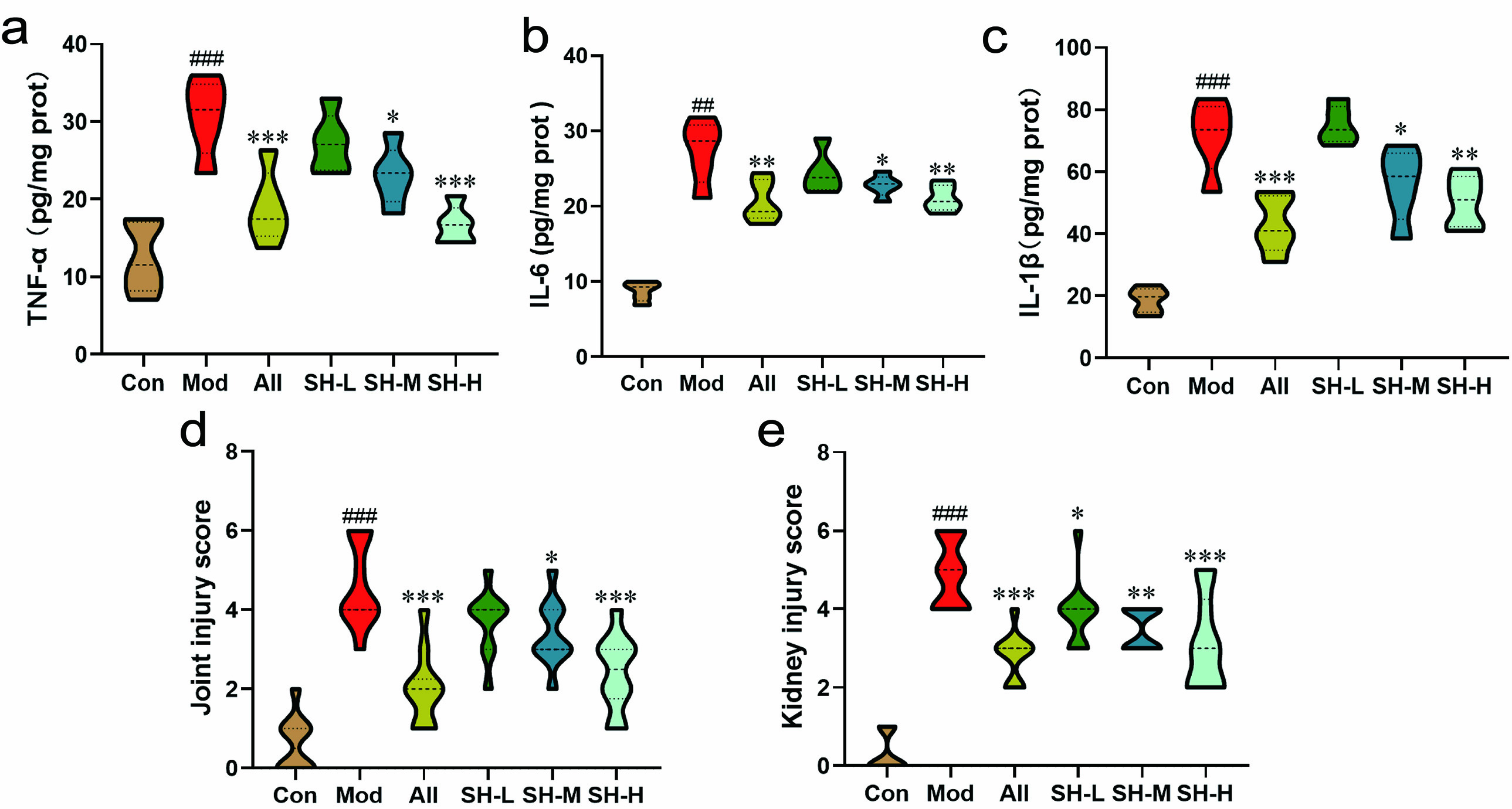


**Fig. S2** Effects of SH-P-1-1 on the inflammatory factors and tissue injuries. (a) Tumor necrosis factor-α (TNF-α); (b) Interleukin-6 (IL-6); (c) Interleukin-1β (IL-1β); (d) Joint injury score; (e) Kidney injury score. Ten randomly selected fields per section were selected and scored. The joint injury was evaluated by inflammatory cell infiltration and synovial cell proliferation, and kidney injury was assessed by inflammatory cell infiltration and renal tubular injury. The scoring criteria were as follows: 0, none; 1, mild; 2, moderate; 3, severe. ##, *P* < 0.01 vs. Con; ###, *P* < 0.001 vs. Con; *, *P* < 0.05 vs. Mod; **, *P* < 0.01 vs. Mod; ***, *P* < 0.001 vs. Mod. n = 5.

**
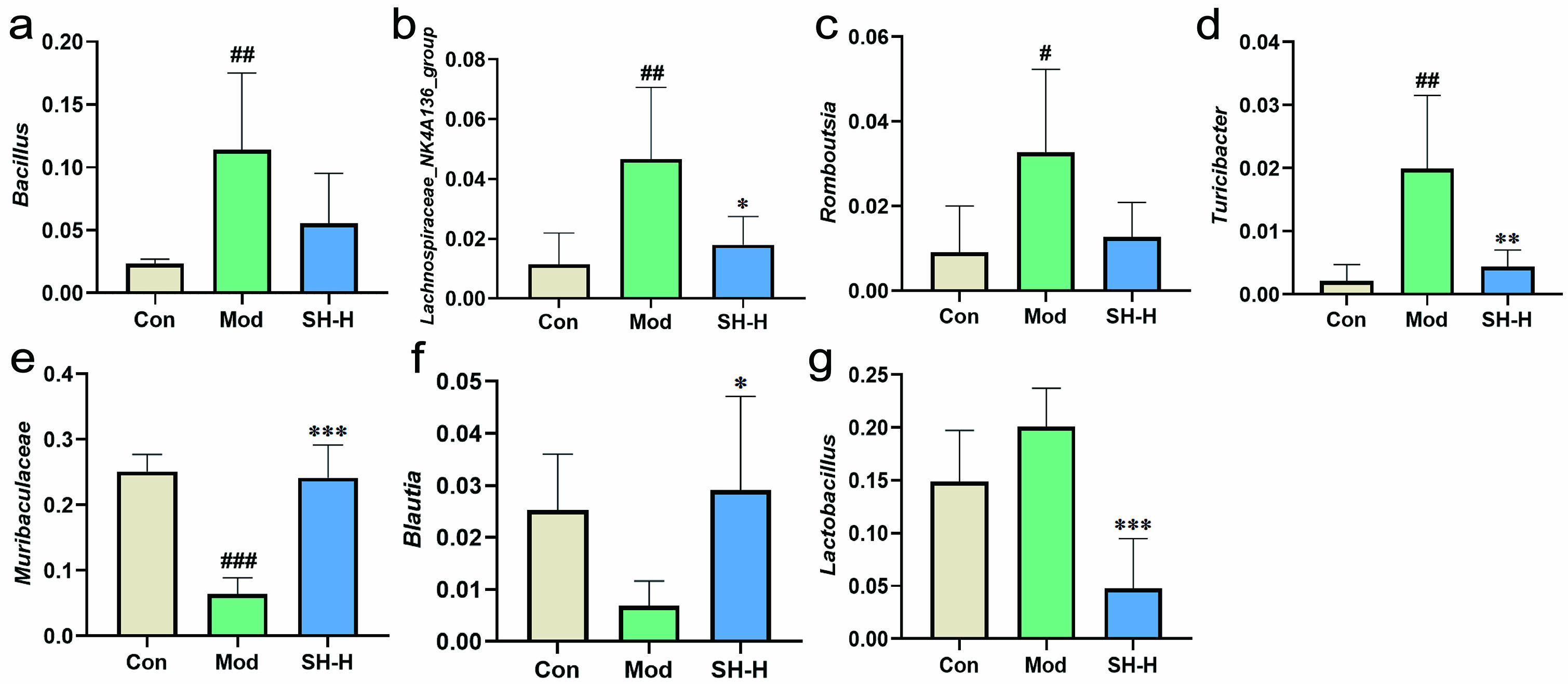
**

**Fig. S3** Relative abundance that exhibited significant differences of the gut microbiota at the genus level. (a) *Bacillus*; (b) *Lachnospiraceae_NK4A136_group*; (c) *Romboutsia*; (d) *Turicibacter*; (e) *Muribaculaceae*; (f) *Blautia*; (g) *Lactobacillus*. #, *P* < 0.05 vs. Con; ##, *P* < 0.01 vs. Con; ###, *P* < 0.001 vs. Con; *, *P* < 0.05 vs. Mod; **, *P* < 0.01 vs. Mod; ***, *P* < 0.001 vs. Mod. n = 5.


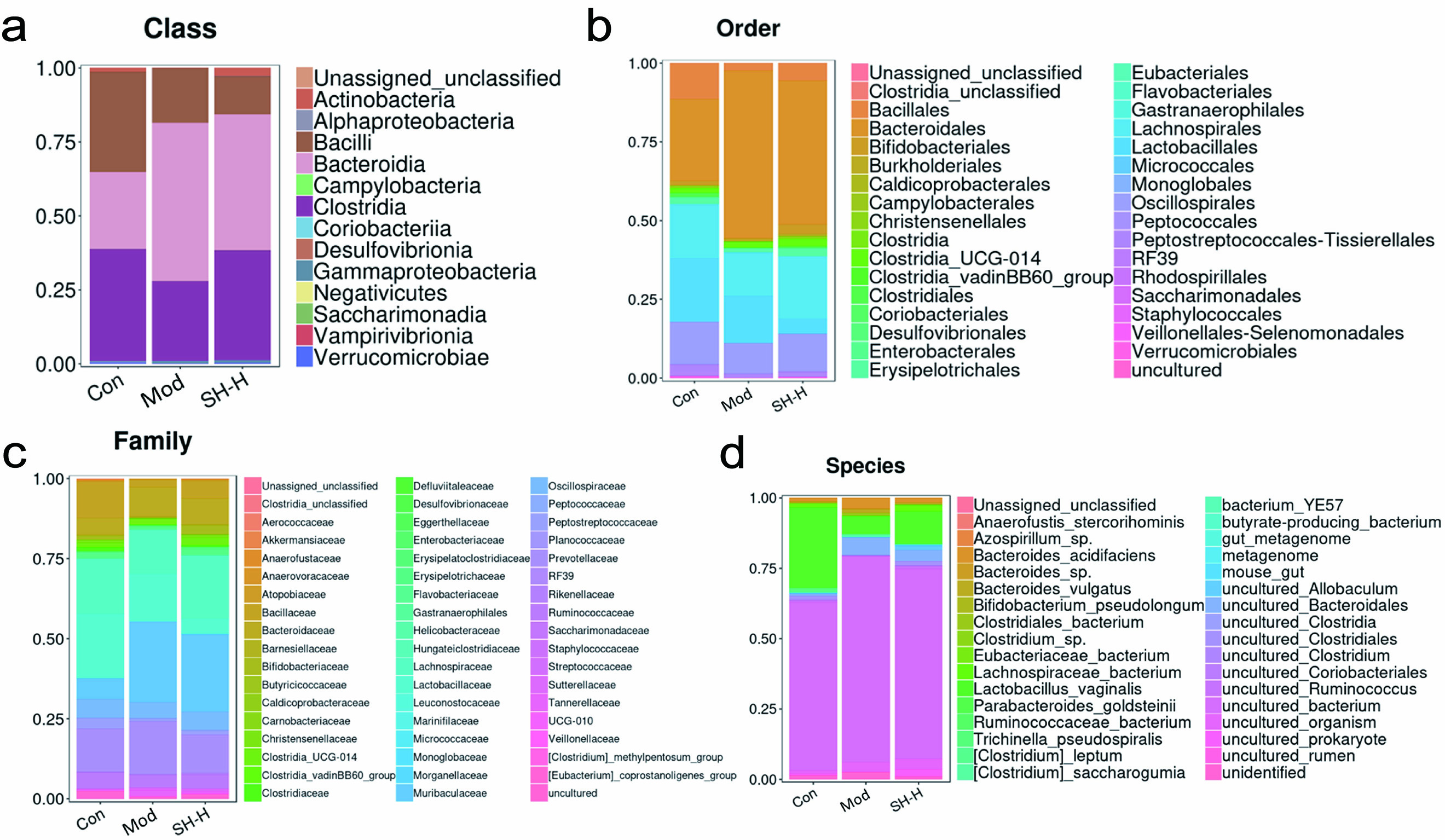


**Fig. S4** Relative abundance of gut microbiota at the (a) class, (b) order, (c) family, and (d) species levels. n = 5.

**
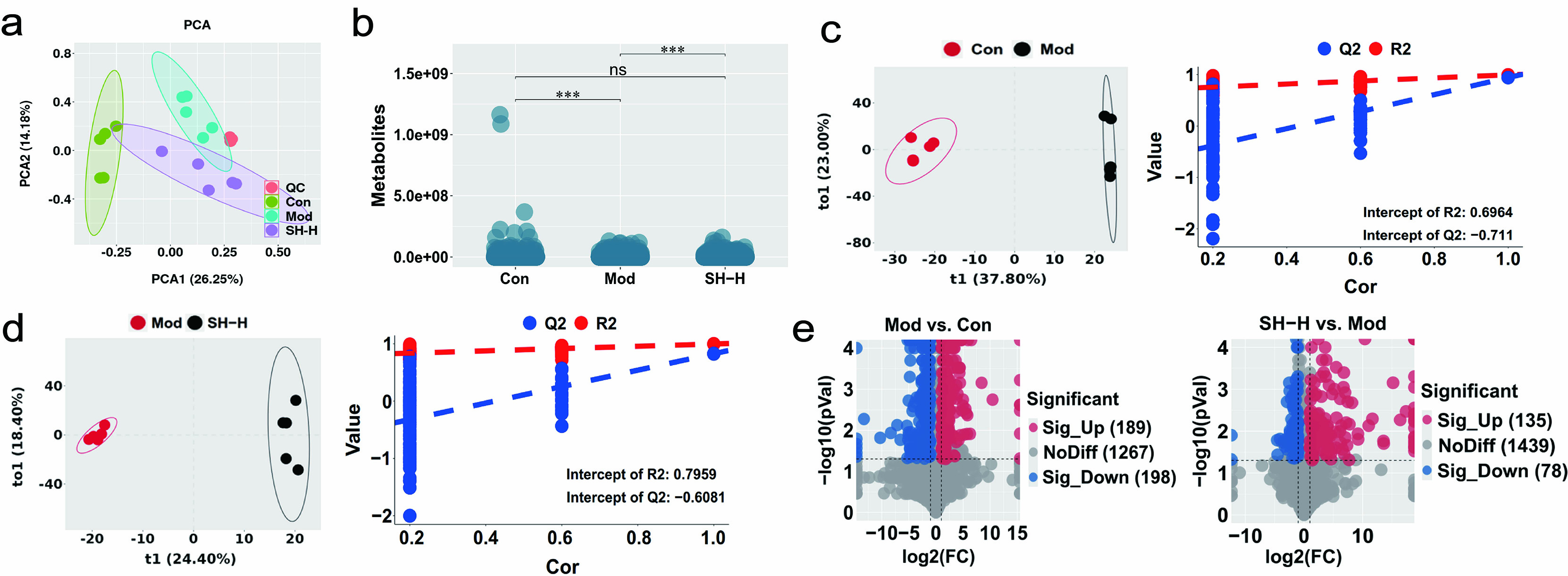
Fig. S5** Untargeted metabolomic analysis in the negtive (NEG) mode. (a) Differences and variation analysis for the samples via principal components analysis (PCA); (b) Differential analysis of metabolites among the different groups; (c) Feature analysis of differential metabolites between the Con and Mod groups via orthogonal projections to latent structures-discriminant analysis (OPLS-DA) and permutation testing; (d) Feature analysis of differential metabolites between Mod and SH-H via OPLS-DA and permutation testing; (e) Differential metabolites between the Con and Mod groups and between the Mod and SH-H groups displayed via a volcano plot. ***, p < 0.001. n = 5.

**
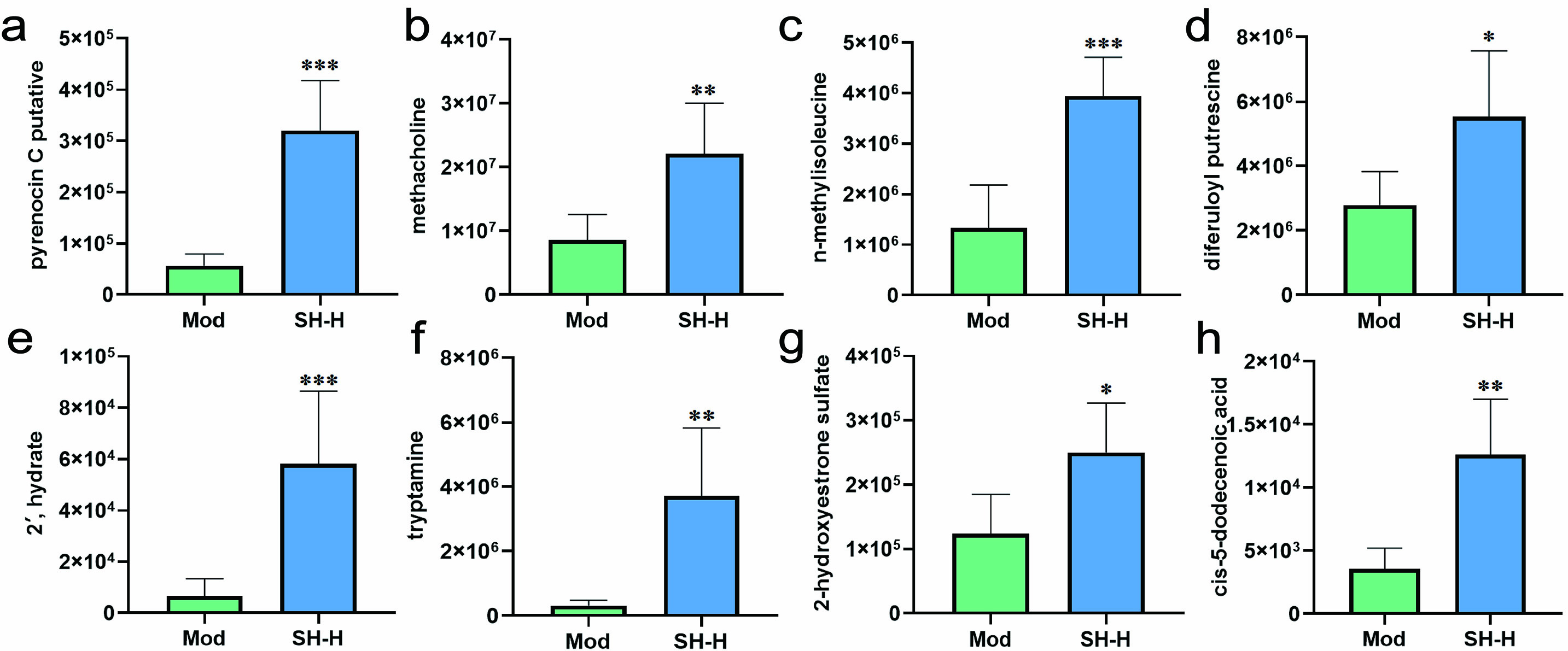
Fig. S6** Differential metabolites that significantly increased after SH-P-1-1 treatment in the postive (POS) mode. (a) pyrenocin C putative; (b) methacholine; (c) n-methylisoleucine; (d) diferuloyl putrescine; (e) 2′, hydrate; (f) tryptamine; (g) 2-hydroxyestrone sulfate; (h) cis-5-dodecenoic acid. *, p < 0.05; **, p < 0.01; ***, p < 0.001. n = 5.


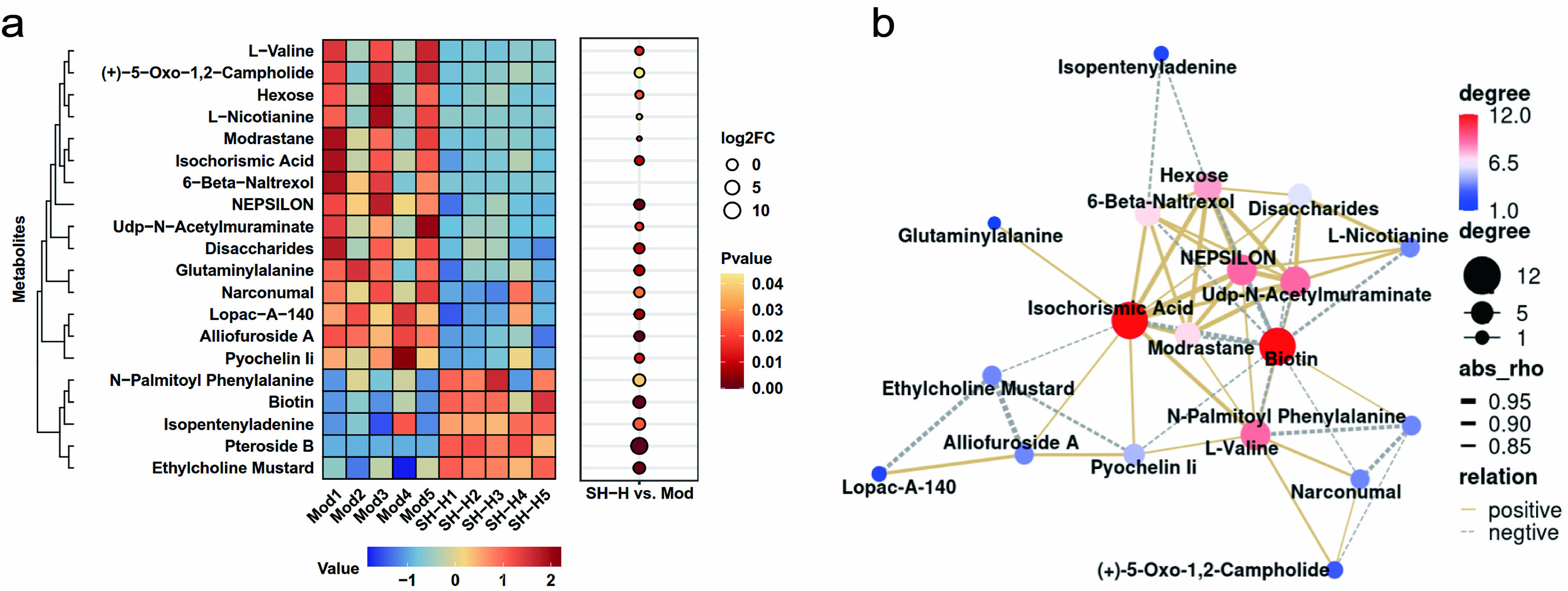
**Fig. S7** Untargeted metabolomic analysis in the negtive (NEG) mode. (a) Hierarchical clustering analysis of differential metabolites between the Mod and SH-H groups (top 20); (b) Correlation analysis of the differential metabolites. n = 5.

**
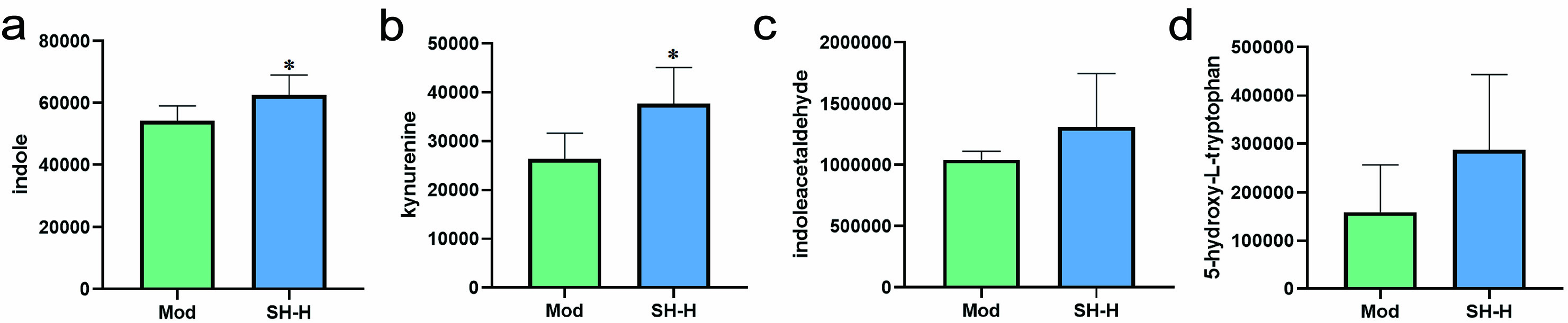
Fig. S8** Change of tryptophan-derived metabolites after SH-P-1-1 treatment in the postive (POS) mode. (a) indole; (b) kynurenine; (c) indoleacetaldehyde; (d) 5-hydroxy-L-tryptophan. *, p < 0.05. n = 5.


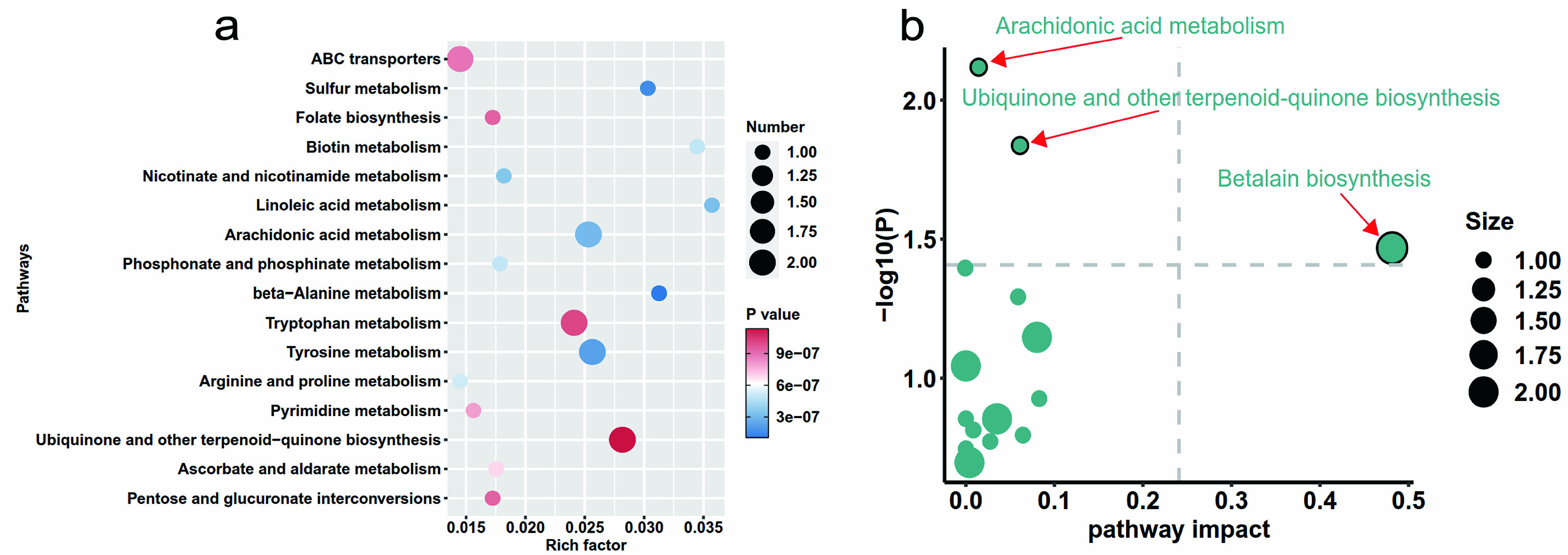
**Fig. S9** Enrichment analysis of the pathways related to the differential metabolites. (a) The key pathways related to the differential metabolites; (b) Kyoto encyclopedia of genes and genomes (KEGG) pathway topology analysis of the differential metabolites. n = 5.


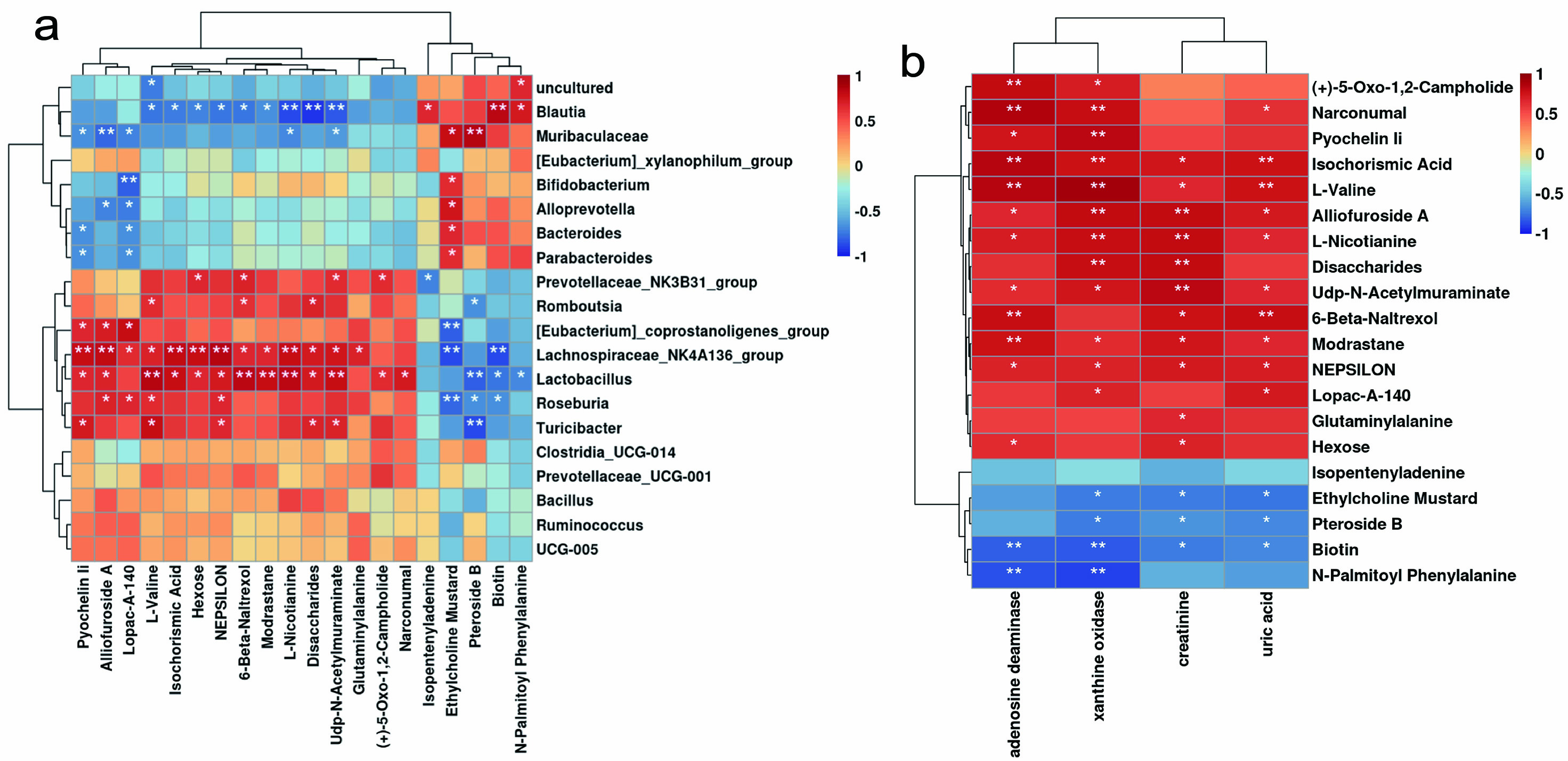
**Fig. S10** Correlation analysis for the intestinal microbiota and differential metabolites in the negtive (NEG) mode between the Mod and SH-H groups. (a) Hierarchical clustering analysis for the relation of the differential metabolites and gut microbiota; (b) Correlation between uric acid (UA), creatinine (CRE), xanthine oxidase (XOD), adenosine deaminase (ADA) and the differential metabolites. *, p < 0.05; **, p < 0.01. n = 5.
